# Phase Separation of NFIB Suppresses SLC3A2‐Mediated Ferroptosis in Castration‐Resistant Prostate Cancer

**DOI:** 10.1002/advs.202515340

**Published:** 2026-03-09

**Authors:** Qiunuo Li, Danyang Chen, Yongzhen Xia, Yili Long, Nan Huang, Rongna Li, Mengting Shi, Mairehaba Kadier, Huafeng Luo, Xiaohui Chen, Hao Liu, Guanmin Jiang

**Affiliations:** ^1^ Department of Clinical Laboratory The Fifth Affiliated Hospital of Sun Yat‐Sen University Zhuhai Guangdong China; ^2^ Affiliated Cancer Hospital & Institute of Guangzhou Medical University Guangzhou Guangdong China

**Keywords:** castration‐resistant prostate cancer, ferroptosis, liquid–liquid phase separation, NFIB, SLC3A2

## Abstract

Castration‐resistant prostate cancer (CRPC) is frequently resistant to conventional therapies and lacks effective treatment options. Although CRPC cells exhibit sensitivity to ferroptosis inducers, the mechanisms regulating ferroptosis remain unclear. Here, we identify nuclear factor I/B (NFIB) as a critical suppressor of ferroptosis in CRPC. NFIB is upregulated in CRPC tissues and cell lines, positively correlating with SLC3A2, a critical subunit of System Xc^−^. NFIB knockout enhances erastin‐induced ferroptosis, marked by elevated Fe^2^^+^, MDA, and ROS levels. Mechanistically, NFIB directly activates SLC3A2 transcription and forms nuclear condensates through intrinsically disordered regions at both the N‐terminus (1–69) and C‐terminus (173–495), with the C‐terminal IDR additionally supporting nuclear localization. Moreover, SIRT7‐dependent deacetylation of NFIB regulates acetylation at K65 within the N‐terminal IDR, thereby tuning condensate dynamics. K65 mutation reduces condensate liquidity and weakens NFIB‐driven SLC3A2 transcriptional activation, resulting in enhanced ferroptosis. In vivo, combined NFIB suppression and ferroptosis induction significantly inhibit tumor growth and increase lipid peroxidation in CRPC xenografts. These findings uncover a critical role of NFIB phase separation and acetylation in ferroptosis regulation and suggest NFIB as a promising therapeutic target in CRPC.

## Graphical abstract

Castration‐resistant prostate cancer (CRPC) remains sensitive to ferroptosis, but its intrinsic resistance is poorly understood. Here, we identify NFIB as a master suppressor. SIRT7‐mediated NFIB acetylation drives its liquid–liquid phase separation, which promotes SLC3A2 transcription to inhibit ferroptosis. Combining NFIB targeting with ferroptosis inducers potently suppresses tumor growth, revealing a promising therapeutic strategy for advanced prostate cancer.

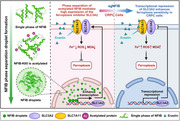

**Abbreviations**
CRPC: castration‐resistant prostate cancer
NFIB: nuclear factor I/B
ADT: androgen‐deprivation therapy
HSPC: hormone‐sensitive prostate cancer
ATCC: American Type Culture Collection
PBS: phosphate‐buffered saline
GSEA: gene set enrichment analysis
ChIP: chromatin immunoprecipitation
Co‐IP: co‐immunoprecipitation
MDA: malondialdehyde
ROS: reactive oxygen species
LLPS: liquid–liquid phase separation
IDR: intrinsically disordered region
FRAP: fluorescence recovery after photobleaching
FSP1: ferroptosis suppressor protein 1
GSH: glutathione
GPX4: glutathione peroxidase 4
TGI: tumor growth inhibition

## 1 Introduction

Prostate cancer is the second most common male malignant tumor in the world, with the first incidence and second mortality among male tumors in Europe and the United States [1, 2]. For men with metastatic prostate cancer, androgen‐deprivation therapy (ADT) is the mainstay of treatment [3]. However, prostate cancer tends to progress from hormone‐sensitive prostate cancer (HSPC) to castration‐resistant prostate cancer (CRPC) after 1–2 years of ADT treatment [4], and there is no reliable method for the treatment of CRPC. Thus, the identification of novel therapeutic targets and intervention strategies is crucial for improving outcomes in CRPC.

Ferroptosis is a regulated form of cell death driven by iron‐dependent lipid peroxidation and excessive accumulation of reactive oxygen species [5]. One of its core mechanisms is the dysfunction of System Xc‐, which consists of SLC3A2 and SLC7A11 and mediates the exchange of extracellular cystine and intracellular glutamate at the plasma membrane, which can be inhibited by ferroptosis induction agent erastin. This in turn triggers iron‐dependent lipid peroxidation and accumulation of lipid peroxides, ultimately leading to ferroptosis of cells [6]. Accordingly, SLC3A2 functions as a critical anti‐ferroptotic factor [7]. Increasing evidence indicates that suppression of SLC3A2 or its associated pathways sensitizes cancer cells to ferroptosis and limits tumor growth across multiple malignancies [8, 9, 10]. The targeted overexpression of SLC3A2 can play an effective anti‐tumor effect in head and neck squamous cell carcinoma [11]. Notably, prostate cancer cells exhibit intrinsic sensitivity to ferroptosis inducers [12], yet the mechanisms by which CRPC cells evade ferroptotic death remain poorly defined, highlighting ferroptosis regulation as a promising therapeutic entry point.

Liquid‐liquid phase separation (LLPS) is a biophysical phenomenon in which phase separation can form membrane‐free organelles in cells, thereby mediating various biological processes [13, 14]. Phase separation is often driven by intrinsic disordered regions (IDRs) and multivalent interactions of proteins, in which post‐translational modifications play a regulatory role [15].Abnormal LLPS also have a certain correlation with tumorigenesis [16, 17]. Ferroptosis‐suppressor‐protein 1 (FSP1) is a second ferroptosis inhibitory system independent of the cysteine‐glutathione (GSH)‐glutathione peroxidase 4 (GPX4) axis. IcFSP1 (a 3‐phenylquinazolinone analogue), as a potent inhibitor of FSP1, was able to promote ferroptosis by facilitating the phase separation of FSP1, suggesting that the phase separation has a role in the regulation of ferroptosis as a potential target in the regulation of ferroptosis [18]. In addition, LLPS‐related gene signatures correlate with ferroptosis activity and prognosis in hepatocellular carcinoma [19]. However, whether and how LLPS directly governs ferroptosis resistance in CRPC remains unknown.

Nuclear factor I/B (NFIB) is a site‐specific DNA‐binding protein. As a transcription factor, NFIB plays an important role in increasing chromatin accessibility and regulating the transcription of a variety of biological processes [20, 21]. We recently reported that NFIB promotes epithelial‐to‐mesenchymal transition and metastasis of CRPC [22]. However, its involvement in ferroptosis regulation remains unexplored. In this study, we investigate the role of NFIB in inhibiting ferroptosis in CRPC. Our findings reveal that NFIB binds to the SLC3A2 promoter and activates its transcription, thereby promoting SLC3A2 expression and inhibiting ferroptosis. We further demonstrate that NFIB undergoes liquid–liquid phase separation, with both the C‐terminal (residues 173–495) and N‐terminal (residues 1–69) IDRs contributing to droplet formation, while the C‐terminal IDR additionally mediates nuclear localization. Both impaired NFIB phase‐separated droplet formation and reduced mobility weaken the ability of NFIB to bind SLC3A2 and inhibits ferroptosis regulation. Furthermore, SIRT7‐regulated acetylation of NFIB at K65 promotes its phase separation, which inhibits ferroptosis by upregulating SLC3A2 expression. Finally, targeting inhibition of NFIB in combination with ferroptosis inducer, such as erastin, significantly suppressed tumor growth, downregulated SLC3A2 expression, increased lipid peroxidation, and accelerated ferroptosis in vivo. Collectively, our study uncovers a phase‐separation–based transcriptional mechanism linking NFIB to ferroptosis resistance and provides a mechanistic rationale for targeting NFIB‐driven pathways in combination with ferroptosis inducers as a therapeutic strategy for CRPC.

## 2 Results

### 2.1 Different Prostate Cancer Cell Types Exhibit Distinct Sensitivity to Ferroptosis

**FIGURE 1:**
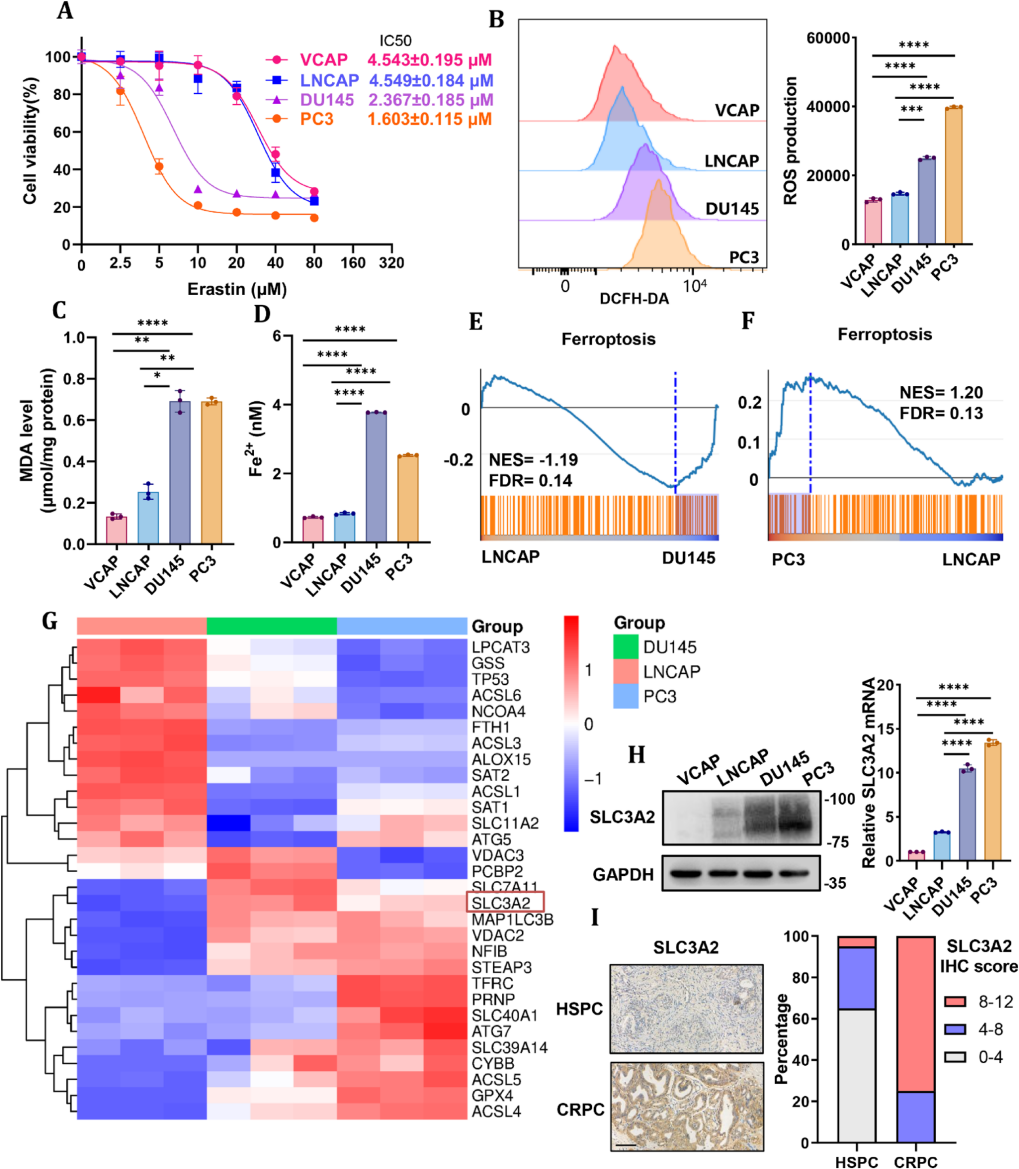
Different prostate cancer cell types exhibit distinct sensitivity to ferroptosis. (A) Cell viability of prostate cancer cell lines (DU145, PC3, VCaP, LNCaP) following treatment with increasing doses of erastin, as assessed by CCK‐8 assay. (B) ROS measurement of prostate cancer cell lines (DU145, PC3, VCaP, LNCaP) following 1 µM erastin treatment for 6 h, and representative images and graph quantifications are shown. (C) MDA level of prostate cancer cell lines (DU145, PC3, VCaP, LNCaP) following 1 µM erastin treatment for 12 h. (D) Fe^2+^ measurement of prostate cancer cell lines (DU145, PC3, VCaP, LNCaP) following 1 µM erastin treatment for 12 h. (E and F) Gene set enrichment analysis (GSEA) analysis of ferroptosis signature in LNCaP, DU145, and PC3 cell lines. (G) Heatmap for ferroptosis pathway regulatory genes and NFIB expression in LNCaP, DU145, and PC3 cell lines. (H) Protein and mRNA expression of SLC3A2 in indicated prostate cancer cell lines. (I) Representative immunohistochemical (IHC) staining and quantification of SLC3A2 protein expression in CRPC specimens (n = 12) and HSPC specimens (n = 20). Scale bar, 100 µm. All quantitative data are presented as mean ± SD from at least three independent biological replicates (n ≥ 3). Statistical significance was determined using a two‐tailed unpaired Student's t‐test for comparisons between two groups or one‐way ANOVA followed by Bonferroni post hoc correction for multiple‐group comparisons. *p ≤ 0.05, **p ≤ 0.01, ***p ≤ 0.001, and ****p ≤ 0.0001.

It has been previously reported that prostate cancer is sensitive to ferroptosis induction [22]. To investigate the differences in sensitivity of different types of prostate cancer cells to ferroptosis induction, we treated prostate cancer cell representing different variants of prostate cancer with different concentrations of erastin in vitro. VCaP and LNCaP cells are characterized with HSPC traits in contrast to DU145 and PC3 with CRPC traits. DU145 and PC3 cells exhibited significantly greater sensitivity to erastin‐induced cell death compared with VCaP and LNCaP cells (Figure 1A). To further characterize ferroptotic responses, we measured intracellular Fe^2^^+^ accumulation, reactive oxygen species (ROS) production, and malondialdehyde (MDA) levels, which are established biochemical hallmarks of ferroptosis. Compared with VCaP and LNCaP cells, DU145 and PC3 cells displayed significantly elevated ROS, MDA, and Fe^2^^+^ levels (Figure 1B–D), indicating the increasing iron load and the lipid peroxidation damage in CRPC cells. Together, these results suggest that prostate cancer cells with CRPC traits are susceptible to ferroptosis induction by erastin.

To explore the reasons for this difference in ferroptosis sensitivity, we performed transcriptomic sequencing on LNCaP, DU145, and PC3 cells. Gene set enrichment analysis (GSEA) revealed that ferroptosis‐related pathways were significantly enriched in DU145 and PC3 cells relative to LNCaP cells (Figure 1E,F). We further analyzed the differences of ferroptosis pathway regulatory genes in each cell (Table S1). Heatmap analysis identified SLC3A2, a key anti‐ferroptotic component of System Xc^−^, as significantly upregulated in CRPC cells (Figure 1G). Western blot and RT‐qPCR showed that the expression levels of SLC3A2 was increased in DU145 and PC3 cells (Figure 1H), and the same trend was found in clinical tissue samples of HSPC and CRPC (Figure 1I). These findings indicate that despite heightened ferroptosis sensitivity, CRPC cells concomitantly activate compensatory anti‐ferroptotic pathways, potentially limiting the efficacy of ferroptosis induction alone.

### 2.2 NFIB Inhibits Erastin‐Induced Ferroptosis in CRPC Cells

**FIGURE 2:**
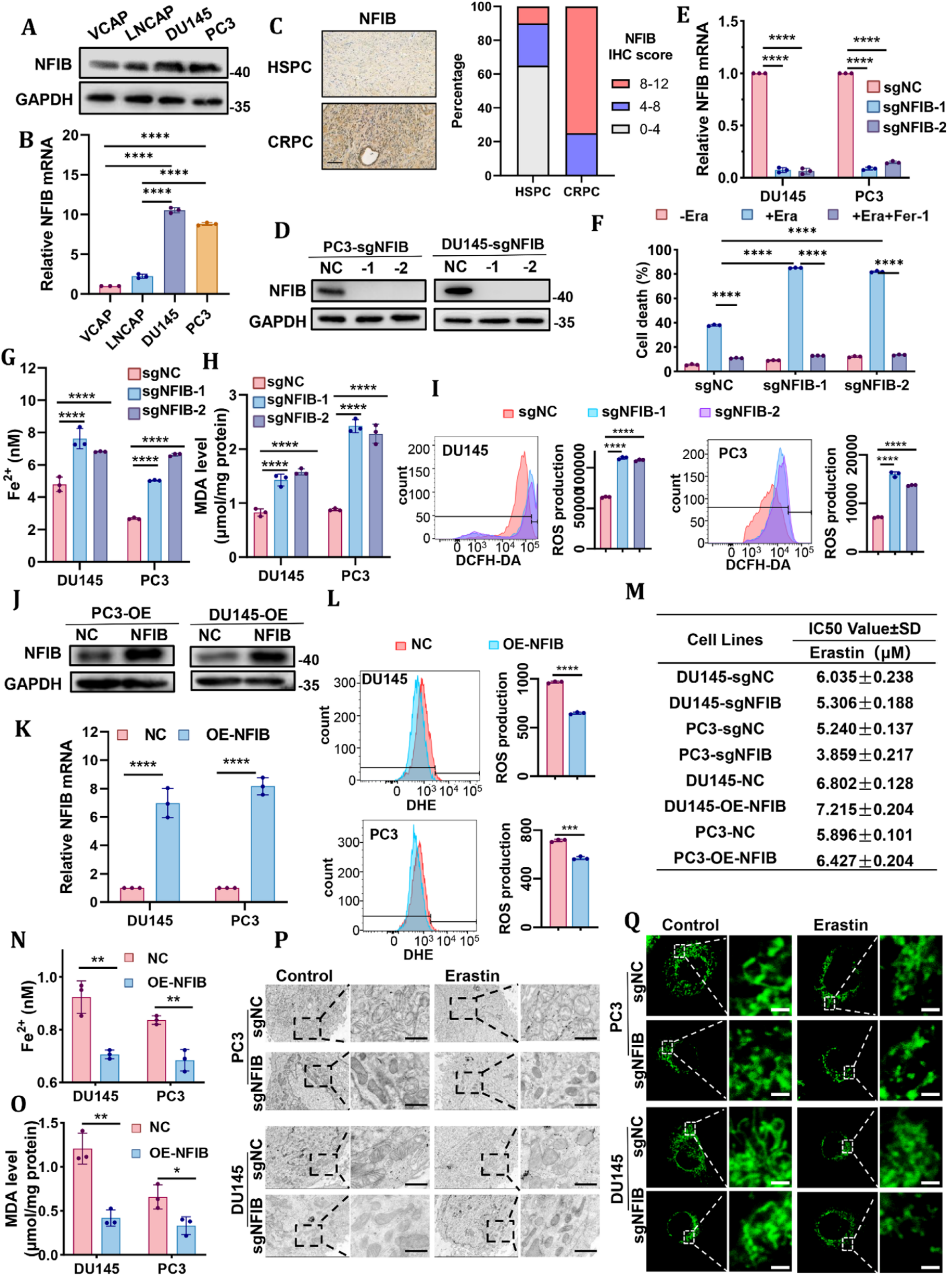
NFIB inhibits erastin‐induced ferroptosis in CRPC cells. (A,B) Protein expression (Western blot) and mRNA levels (RT–qPCR) of NFIB in prostate cancer cell lines (DU145, PC3, VCaP, LNCaP). (C) Representative IHC staining and quantification of NFIB protein expression in CRPC specimens (n=12) and HSPC specimens (n=20). The scale bar represents 100 µm. (D,E) Validation of NFIB knockout efficiency by Western blot and RT–qPCR in in cells with stable knockout of NFIB with 2 independent clones. (F) Cell death asssay in control or NFIB stable knockout DU145 with 2 independent clones treated with erastin in the presence or absence of fer‐1. (G) Intracellular Fe^2^^+^ levels in control or NFIB stable knockout DU145 and PC3 cells with 2 independent clones following erastin treatment. (H) MDA level of NFIB stable knockout DU145 and PC3 cells with 2 independent clones following erastin treatment. (I) ROS measurement of control or NFIB stable knockout DU145 and PC3 cells with 2 independent clones following erastin treatment, and representative images and graph quantifications are shown. (J,K) Protein expression and mRNA of NFIB was performed on DU145‐OE‐NFIB and PC3‐OE‐NFIB cells. (L) ROS measurement of DU145‐OE‐NFIB and PC3‐OE‐NFIB cells following erastin treatment, and representative images and graph quantifications are shown. (M) Cell viability was assessed by CCK‐8 assay after treatment with different concentrations of erastin in corresponding prostate cancer cells and IC50 were listed. (N,O) MDA level and Fe^2+^ measurement of DU145‐OE‐NFIB and PC3‐OE‐NFIB cells following erastin treatment. (P) NFIB‐depleted DU145 and PC3 cells were treated with erastin and analyzed by TEM. The scale bar represents 1 µm. (Q) Mitochondrial morphology was visualized by MitoTracker staining in NFIB‐depleted DU145 and PC3 cells treated with or without erastin. Scale bar, 2 µm. All quantitative data are presented as mean ± SD from at least three independent biological replicates (n ≥ 3). Differences between groups were assessed using a two‐tailed unpaired Student's t‐test, or one‐way ANOVA test with Bonferroni's correction. *p ≤ 0.05, **p ≤ 0.01, ***p ≤ 0.001, and ****p ≤ 0.0001.

In previous studies, we have found that the key transcription factor NFIB is highly expressed in DU145 and PC3 cells and is highly correlated with EMT and metastasis [12]. Consistent with these findings, NFIB expression was significantly elevated in DU145 and PC3 cells, and a similar trend was observed in clinical CRPC tissues (Figure 2A–C). Importantly, we found that SLC3A2, which is negative regulator of ferroptosis, was expressed at higher protein levels in DU145 and PC3 cells compared to VCaP and LNCaP cells (Figure 1H). These results suggest that the high expression of NFIB in CRPC cells may be associated with ferroptosis regulation.

To further explore the potential role of NFIB on ferroptosis in CRPC cells, DU145 and PC3 cell lines with stable knockout of NFIB were constructed using CRISPR‐Cas9 with 2 independent clones, along with their respective control cells (Figure 2D,E), designated as DU145‐sgNFIB/DU145‐sgNC and PC3‐sgNFIB/PC3‐sgNC. To clarify the relationship between NFIB and ferroptosis, DU145‐sgNFIB/DU145‐sgNC cells were treated with the ferroptosis inducer erastin and the ferroptosis inhibitor Fer‐1, followed by detection of the cell death rate. The results indicated that erastin increased the rate of dead cells, while Fer‐1 significantly alleviated cell death, with this effect being more pronounced in NFIB‐deficient cells (Figure 2F; Figure S1I). A similar trend was observed in PC3‐sgNFIB/PC3‐sgNC cells, indicating that NFIB loss enhances erastin‐induced ferroptosis. Importantly, combined NFIB knockout and erastin treatment resulted in a greater reduction in cell viability than either intervention alone, suggesting an enhanced ferroptotic response.

The effect of NFIB knockout on ferroptosis in CRPC cells treated with different doses of erastin was further quantitatively analyzed. The results showed that DU145 and PC3 cells exhibited increased susceptibility to erastin‐induced ferroptosis compared with control cells (Figure 2M). The dose–response curves of NFIB‐deficient cells exhibited a pronounced leftward shift, accompanied by a significant reduction in IC_50_ values, indicating a broadly enhanced and dose‐dependent sensitivity to erastin‐induced ferroptosis compared with control cells. Moreover, NFIB depletion consistently resulted in a significant reduction in cell viability across the tested erastin concentration range, indicating a broadly enhanced sensitivity to erastin‐induced ferroptosis. Importantly, erastin at concentrations of 5–10 µM produced the most robust and reproducible differences in cell viability between NFIB‐manipulated cells and their respective controls, while avoiding overt nonspecific cytotoxicity (Figure S1C,D). Based on these observations, 10 µM erastin was selected as the standard working concentration for subsequent mechanistic and biochemical analyses of ferroptosis. In addition, after erastin treatment, CRPC cells showed aggravated iron loading and increased lipid peroxidation damage after NFIB knockout (Figure 2G–I). In contrast, NFIB overexpression rendered CRPC cells resistant to erastin‐induced ferroptosis, as evidenced by increased cell viability together with reduced iron accumulation and lipid peroxidation under identical treatment conditions (Figure 2J–O; Figure S1E–H). Interestingly, NFIB knockout combined with erastin treatment in CRPC cells induced mitochondrial shrinkage, increased membrane density, and disrupted cristae structure, which are typical morphologic features of ferroptosis (Figure 2P,Q). These results indicated that NFIB functions as a negative regulator of erastin‐induced ferroptosis in CRPC cells.

### 2.3 NFIB Directly Binds to the SLC3A2 Promoter Region and Promotes SLC3A2 Transcription

**FIGURE 3:**
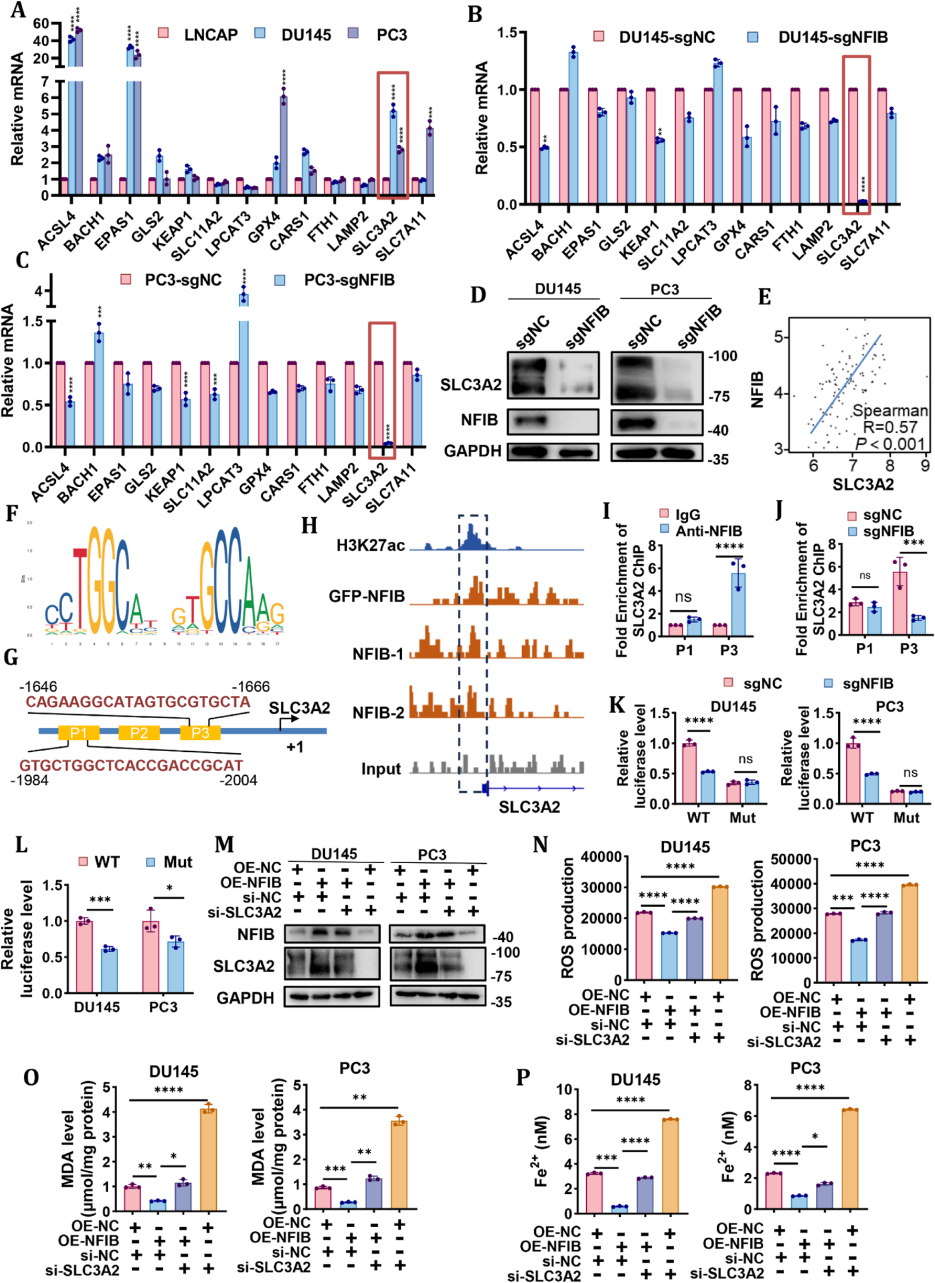
NFIB directly binds to the SLC3A2 promoter region and promotes SLC3A2 transcription. (A) Relative mRNA of ferroptosis regulatory genes on prostate cancer cell lines (DU145, PC3, LNCaP). (B,C) Relative mRNA of ferroptosis regulatory genes on DU145‐sgNFIB and PC3‐sgNFIB cells. (D) Protein expression of SLC3A2 was performed on DU145‐sgNFIB and PC3‐sgNFIB cells. (E) Correlation analysis of NFIB and SLC3A2 expression in prostate cancer tissues using the GEPIA database. (F) Putative NFIB‐binding sites identified in the promoter region of SLC3A2 gene. (G) Schematic diagram of the primer pair location in the SLC3A2 promoter and the corresponding sequences. (H) Integrative Genomics Viewer (IGV) tracks of NFIB ChIP‐seq data showing NFIB binding peaks at the SLC3A2 genomic locus. (I) ChIP‐qPCR of NFIB binding to P1 and P3 on the SLC3A2 promoter in DU145 cells, with IgG as a negative control. (J) ChIP‐qPCR of NFIB binding to P1 and P3 on the SLC3A2 promoter in control or NFIB stable knockout DU145 cells. (K) SLC3A2 promoter activity was measured by dual luciferase reporter assays after transfection with wild‐type and mutant reporter in DU145 and PC3 cells. (L) SLC3A2 promoter activity was measured by dual luciferase reporter assays after transfection with wild‐type and mutant reporter in control or NFIB stable knockout DU145 and PC3 cells. (M) Protein expression in DU145 and PC3 cells overexpressing NFIB or control vector, combined with knockdown of SLC3A2 or control. (N) ROS production in DU145 and PC3 cells overexpressing NFIB or control vector, combined with knockdown of SLC3A2 or control. (O) MDA levels in DU145 and PC3 cells overexpressing NFIB or control vector, combined with knockdown of SLC3A2 or control. (P) Fe^2^^+^ in DU145 and PC3 cells overexpressing NFIB or control vector, combined with knockdown of SLC3A2 or control. The data were expressed as mean ± standard deviation from at least three independent experiments. Differences between groups were assessed using a two‐tailed unpaired Student's *t*‐test, or one‐way ANOVA test with Bonferroni's correction. *p ≤ 0.05, **p ≤ 0.01, ***p ≤ 0.001 and ****p ≤ 0.0001.

In order to investigate the mechanism of NFIB regulating ferroptosis in CRPC cells, mRNA expression levels of 13 ferroptosis regulators were examined. RT‐qPCR analysis revealed significantly higher SLC3A2 expression in CRPC‐derived DU145 and PC3 cells compared to hormone‐sensitive LNCaP cells (Figures 3A and 1H). Notably, SLC3A2 expression was markedly reduced following NFIB knockout (Figure 3B–D). In contrast to SLC3A2, NFIB knockout did not decrease significantly on the expression of SLC7A11, which forms System Xc‐ with SLC3A2. Furthermore, transcriptomic analysis using GEPIA (http://gepia.cancer‐pku.cn/) revealed a significant positive correlation between NFIB and SLC3A2 expression in prostate cancer tissues (Figure 3E). Based on JASPAR (https://jaspar.elixir.no/) and UCSC (https://genome‐asia.ucsc.edu/index.html) databases, we predicted the possible NFIB promoter binding sites on SLC3A2 (Figure 3F,G). The prediction of NFIB binding sites in the SLC3A2 promoter using the JASPAR website revealed two putative motifs P1 and P3 in the SLC3A2 promoter. Chromatin immunoprecipitation (ChIP) sequencing data of NFIB were analyzed to examine the distribution of NFIB binding sites in the genome of HeLa cells [23]. ChIP‐seq showed that there were NFIB binding peaks in the promoter region of the SLC3A2 gene (Figure 3H).

Next, we further elucidated the transcriptional regulation of NFIB in SLC3A2 expression. ChIP–qPCR was designed to confirm the NFIB‐binding site in the SLC3A2 promoter region, and the results confirmed that the P3 site of the promoter region might be the binding site for NFIB rather than P1 (Figure 3I,J; Table S2). To determine whether the NFIB binding site in the P3 promoter region could regulate the transcription of SLC3A2 gene, the promoter region of SLC3A2 gene and its mutant sequence were constructed and luciferase reporter vector was constructed. The luciferase reporter gene assay results showed that the luciferase promoter activity affected the expression of luciferase after the NFIB binding site mutation (Figure 3L). In addition, NFIB knockout decreased wild‐type SLC3A2 promoter reporter activity, while mutant promoter activity remained unaffected (Figure 3K,L). To determine whether NFIB‐driven ferroptosis resistance is mediated by SLC3A2, DU145 and PC3 cells stably overexpressing NFIB were transduced with knockdown of SLC3A2. Western Blot showed that the overexpression of NFIB increased the expression of SLC3A2, while SLC3A2 knockdown did not affect the expression level of NFIB, indicating that NFIB is the upstream of SLC3A2 (Figure 3M). The measurements of ROS, MDA and Fe^2^^+^ revealed that NFIB overexpression suppressed ferroptosis, whereas concomitant SLC3A2 knock‐down fully reversed this effect, restoring high ROS, MDA and Fe^2^^+^ levels (Figure 3N–P). Similarly, SLC3A2 depletion alone was sufficient to trigger ferroptosis without altering NFIB protein expression (Figure 3M–P). Collectively, these data demonstrate that NFIB acts upstream of SLC3A2 and that its ability to suppress ferroptosis is largely dependent on SLC3A2 activity. Considering that SLC3A2 and SLC7A11 together constitute the System Xc^−^, which is responsible for cystine import and subsequent GSH synthesis, GSH levels were measured in CRPC cells. The results showed that NFIB knockout decreased intracellular GSH levels, whereas NFIB overexpression increased them. SLC3A2 knockdown in NFIB‐overexpressing cells lowered GSH levels relative to NFIB overexpression alone, and SLC3A2 knockdown alone also reduced GSH levels (Figure S2A,B). Thus, SLC3A2 downregulation impairs System Xc^−^ function and decreases GSH, indicating that NFIB regulates GSH synthesis and ferroptosis sensitivity via SLC3A2. Taken together, these data suggest that NFIB stimulates SLC3A2 transcription by interacting directly with the SLC3A2 promoter region.

### 2.4 NFIB Undergoes Liquid–Liquid Phase Separation in CRPC Cells

**FIGURE 4:**
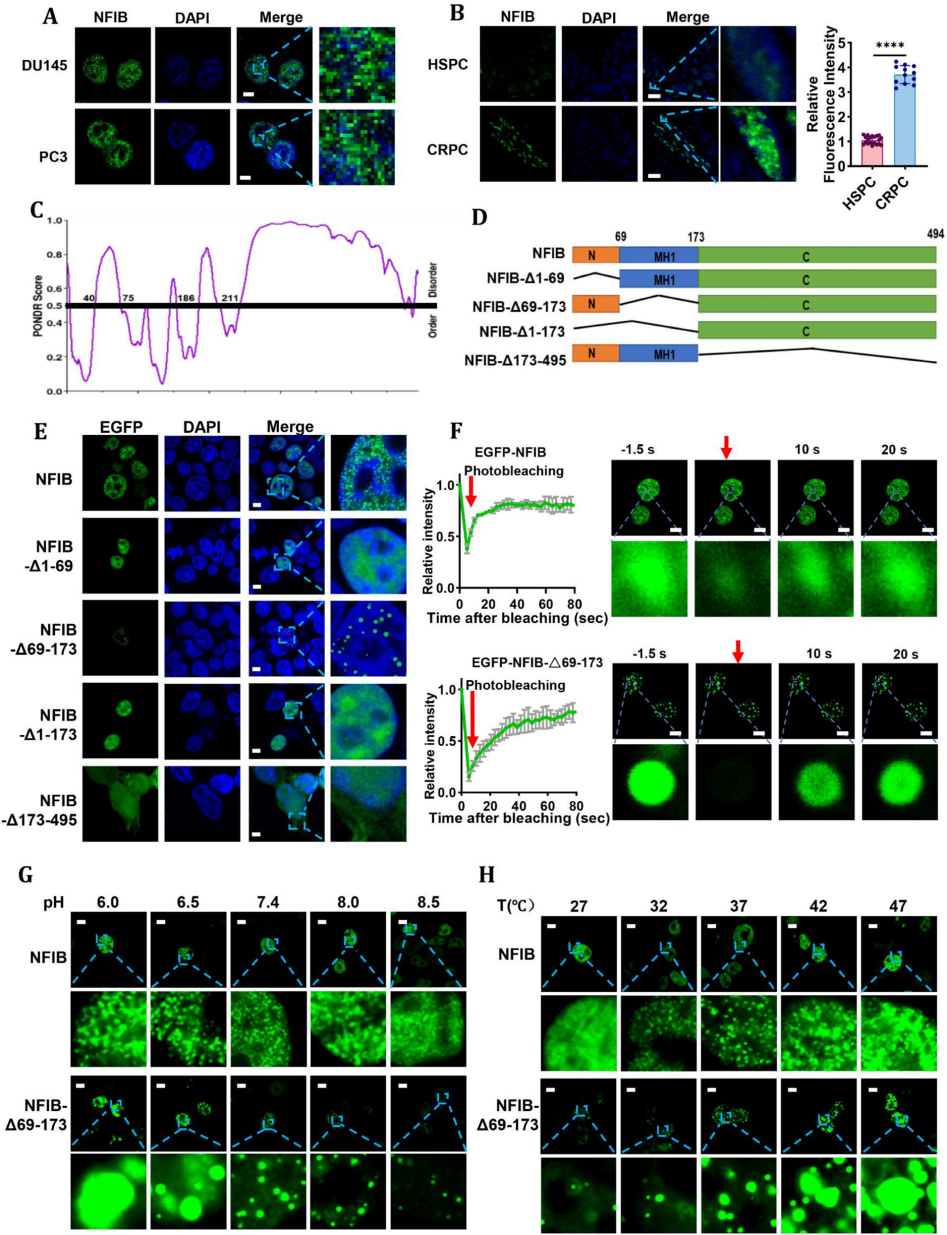
NFIB has the ability of phase separation in CRPC cells. Representative images of NFIB in DU145 and PC3 cells. Scale bar, 10 µm. Representative images of NFIB expression in clinical tissue samples from HSPC (n = 20) and CRPC patients (n = 12). Scale bar, 20 µm. (C) Graph plotting IDRs of NFIB using PONDR. (D) Schematic representation of NFIB and its mutants. (E) Representative image of forming NFIB segment deficient droplets in PC3 cells. Scale bar, 10 µm. (F) FRAP recovery curves (the left panel) and representative images of EGFP‐NFIB and deletion mutants (the right panel) in PC3 cells following photobleaching. Scale bar, 10 µm. (G) Growth and fusion of NFIB droplets at different Ph values in PC3 cells. Scale bar, 10 µm. (H) Growth and fusion of NFIB droplets at different temperatures in PC3 cells. Scale bar, 10 µm. The data were expressed as mean ± standard deviation from at least three independent experiments. Differences between groups were assessed using a two‐tailed unpaired Student's t‐test, or one‐way ANOVA test with Bonferroni's correction. *p ≤ 0.05, **p ≤ 0.01, ***p ≤ 0.001, and ****p ≤ 0.0001.

To further explore the mechanism by which NFIB regulates ferroptosis in CRPC cells, we examined the distribution of endogenous NFIB in DU145 and PC3 cells. Confocal microscopy revealed that NFIB was unevenly distributed within the nucleus and formed punctate structures, which are consistent with phase‐separated nuclear condensates (Figure 4A), suggesting that NFIB may possess the capacity to undergo liquid–liquid phase separation. Immunofluorescence analysis of clinical prostate cancer specimens further confirmed that NFIB was markedly upregulated in CRPC compared with HSPC tissues and exhibited a prominent droplet‐like nuclear distribution (Figure 4B). Consistent with these findings, similar nuclear punctate patterns of NFIB were observed in CRPC xenograft tumors in mice (Figure S3A) [24], supporting the in vivo association of NFIB condensation with advanced disease.

Using the PONDR prediction tool (https://www.pondr.com/), we identified IDRs at both the N‐terminus and the C‐terminus of NFIB (Figure 4C). Proteins harboring IDRs are typically prone to phase separation under physiological conditions. NFIB is mainly composed of two distinct structural modules: an N‐terminal highly conserved DNA‐binding/dimerization domain (MH1) and a C‐terminal transcription modulation region, plus a conserved pre‐N‐terminus region (8–47 aa) with unknown function. To test the necessity of each IDR and structural module for NFIB phase separation, we generated the following EGFP‐tagged deletion mutants (Figure 4D and Supplemental information 1): EGFP‐NFIBΔ1‐69, which deletes the pre‐N‐terminus IDR (residues 1–69); EGFP‐NFIBΔ69‐173, which removes the MH1 DNA‐binding/dimerization domain (residues 69–173); EGFP‐NFIBΔ1‐173, lacking the entire pre‐N‐terminal IDR and MH1 module (residues 1–173); EGFP‐NFIBΔ173‐495, deleting the C‐terminal transcriptional modulation region (residues 173–495) [25]. These constructs were transfected into PC3 cells, and NFIB condensate formation was systematically analyzed. Deletion of the C‐terminal region (EGFP‐NFIBΔ173–495) completely abolished NFIB droplet formation and resulted in pronounced nuclear export, indicating that the C‐terminal IDR is indispensable for condensate nucleation and nuclear retention. In contrast, EGFP‐NFIBΔ1–69 and EGFP‐NFIBΔ1–173 mutants failed to form phase‐separated droplets but remained predominantly nuclear, suggesting that the N‐terminal IDR is dispensable for nuclear localization but required for efficient condensate assembly. Notably, EGFP‐NFIBΔ69–173 retained robust nuclear droplet formation, demonstrating that the MH1 DNA‐binding/dimerization domain is not required for NFIB phase separation (Figure 4E). To biochemically validate these observations, cytoplasmic and nuclear fractionation followed by Western blot analysis was performed to assess the subcellular localization of NFIB deletion mutants. Consistent with fluorescence microscopy results, all NFIB constructs were predominantly localized in the nuclear fraction, except for EGFP‐NFIBΔ173–495, which showed substantial cytoplasmic distribution (Figure S3B). These results provide independent biochemical confirmation that the C‐terminal IDR is essential for NFIB nuclear retention and condensate formation. We next evaluated the liquidity and dynamic properties of NFIB condensates using fluorescence recovery after photobleaching (FRAP). Because only EGFP‐NFIB and EGFP‐NFIBΔ69–173 formed discernible nuclear condensates, FRAP analysis was restricted to these droplet‐forming constructs. Both EGFP‐NFIB and EGFP‐NFIBΔ69–173 exhibited rapid and efficient fluorescence recovery, indicative of high molecular mobility within the condensates and liquid‐like behavior (Figure 4F). Taken together with the loss of condensate formation observed upon N‐ or C‐terminal IDR deletion, these findings support the conclusion that while the C‐terminal IDR is essential for droplet nucleation, the N‐terminal IDR (residues 1–69) is required to enable functional, dynamic phase separation of NFIB.

To further characterize the biophysical properties governing NFIB liquid–liquid phase separation, we attempted recombinant expression of full‐length NFIB for in vitro reconstitution assays. However, owing to the extensive intrinsically disordered regions, NFIB proved difficult to express and purify in a soluble form, precluding reliable in vitro droplet assays. We therefore assessed the environmental sensitivity of NFIB condensates using live‐cell imaging of fluorescently labeled NFIB constructs, an approach widely used to characterize phase separation in cellular contexts. Live‐cell imaging revealed that increasing extracellular pH led to a progressive dissolution of NFIB condensates, with droplets becoming markedly reduced and more diffuse under alkaline conditions (pH 8.5) (Figure 4G). Conversely, elevated temperature (27°C‐47°C) significantly enhanced both the formation and growth of NFIB condensates (Figure 4H), indicating temperature‐dependent phase behavior. These observations demonstrate that NFIB condensates exhibit pronounced environmental tunability, a defining feature of liquid‐like biomolecular assemblies. Furthermore, treatment with 1,6‐hexanediol (1,6‐HD), a reagent known to disrupt weak hydrophobic interactions, resulted in rapid dissolution of NFIB condensates (Figure S3C), supporting the conclusion that these structures are maintained by reversible, low‐affinity interactions characteristic of phase‐separated condensates.

Taken together, these results provide convergent evidence that NFIB undergoes LLPS in the nucleus, characterized by dynamic molecular exchange, sensitivity to physicochemical conditions, and dependence on intrinsically disordered regions.

### 2.5 Acetylation at K65 Within the N‐Terminal IDR of NFIB Regulates Its Phase Separation

**FIGURE 5:**
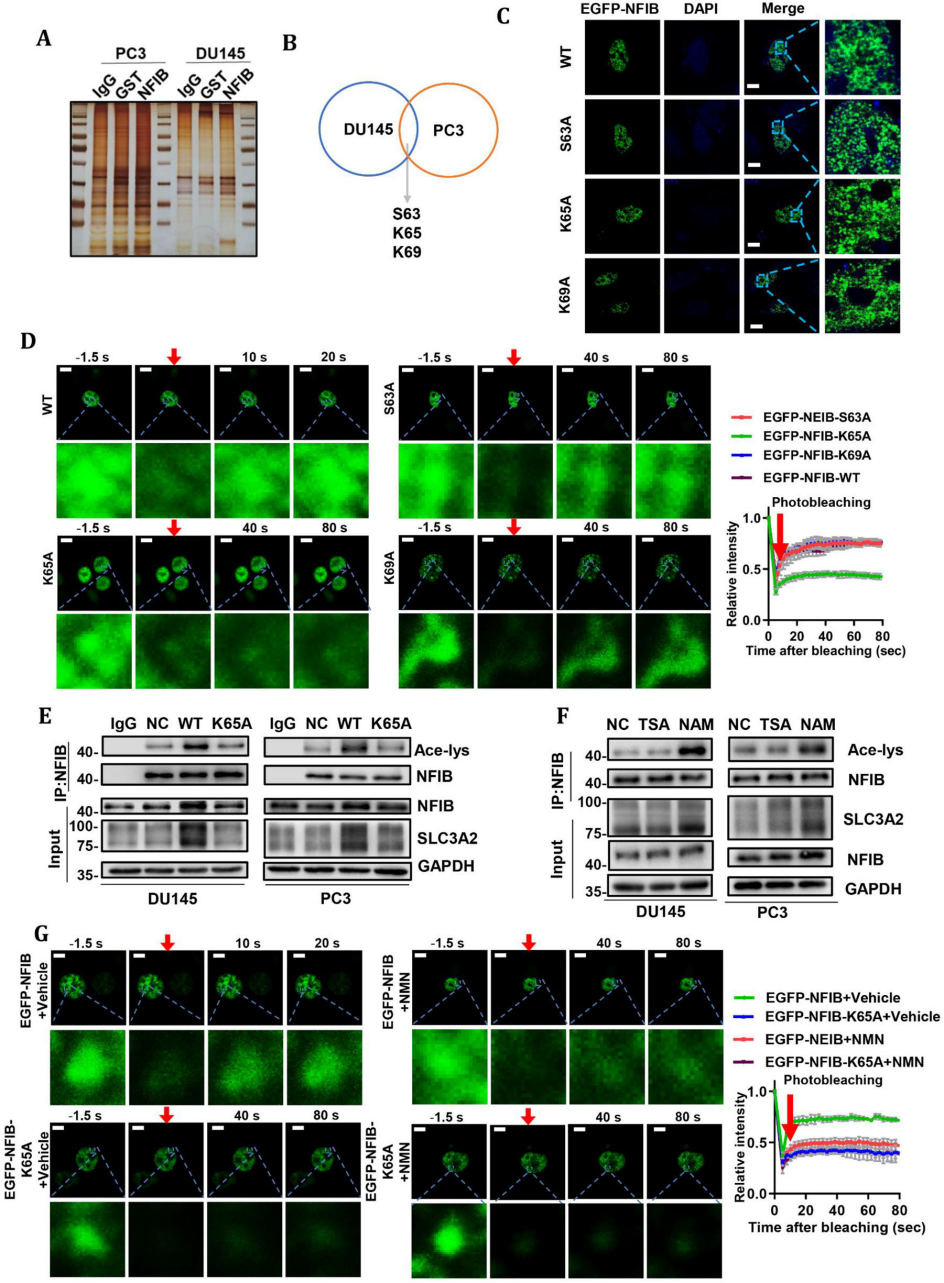
Acetylation at K65 regulates NFIB phase separation. (A) Silver staining showing the IP of NFIB. (B) Schematic representation of intersections among the NFIB post‐translational modifications detected in DU145 and PC3 cells. (C) Representative images of mutations at different sites of EGFP‐NFIB in DU145 cells. Scale bar, 10 µm. (D) Representative images and FRAP curve of mutations at different sites of EGFP‐NFIB in PC3 cells. The red arrow indicates the action of bleaching. Scale bar, 10 µm. (E) Acetylation of K65A mutants in DU145 and PC3 cells with NFIB knockout. (F) Acetylation of NFIB in DU145 and PC3 cells treated with deacetylase inhibitors TSA or NAM. Input showed the protein expression of SLC3A2. (G) Time course analysis of K65A treated with NMN recovery after photobleaching in PC3 cells. Representative images of fluorescence recovery are shown. Scale bar, 10 µm. The data were expressed as mean ± standard deviation from at least three independent experiments. Differences between groups were assessed using a two‐tailed unpaired Student's t‐test, or one‐way ANOVA test with Bonferroni's correction. *p ≤ 0.05, **p ≤ 0.01, ***p ≤ 0.001 and ****p ≤ 0.0001.

Having established that NFIB undergoes liquid–liquid phase separation in CRPC cells and that its N‐terminal IDR is required for maintaining condensate dynamics, we next sought to determine how NFIB phase separation is regulated at the post‐translational level. Thus, we performed immunoprecipitation (IP) of endogenous NFIB from CRPC cells, followed by mass‐spectrometry analysis of post‐translational modifications. By intersecting the modification profiles from both cell lines (Figure 5A,B), we identified three key modifications within the N‐terminal IDR (residues 1–69): phosphorylation at S63, acetylation at K65, and ubiquitination at K69 (Supplemental information 2). Therefore, we generated EGFP‐tagged NFIB mutants in the pReceiver‐Lv201 vector, and mutated the amino acid residue corresponding to the site to Ala, which were S63A, K65A, and K69A (Supplemental information 1). All constructs formed nuclear puncta comparable to wild‐type NFIB when expressed in PC3 cells, indicating that none of these single‐site mutations abrogate droplet nucleation (Figure 5C). Moreover, FRAP analysis revealed a selective impairment of droplet fluidity in the K65A mutant, with significantly slower fluorescence recovery compared to wild‐type NFIB, whereas the S63A and K69A mutants displayed recovery kinetics indistinguishable from wild‐type (Figure 5D). These results demonstrate that acetylation at K65, but not phosphorylation at S63 or ubiquitination at K69, is essential for maintaining the dynamic liquidity of NFIB condensates, thereby pinpointing K65 as a critical regulatory hub for the phase separation of NFIB function.

Furthermore, we investigated whether the acetylation modification at this site was critical for ferroptosis. Co‐IP experiments showed that NFIB was acetylated in CRPC cells (Figure S3D). Moreover, the acetylation level of NFIB decreased after mutation at K65 (Figure 5E). Deacetylases that regulate acetylation are mainly divided into two families, namely HDAC family and SIRT family [26]. To explore which family is responsible for the acetylation modification of NFIB, CRPC cells were treated with the respective inhibitors TSA (HDAC family) and NAM (SIRT family). IP results showed that compared with the control group, the acetylation level of NFIB was increased after NAM treatment, while TSA treatment did not change the acetylation level of NFIB (Figure 5F). Additionally, to investigate whether acetylation at K65 influences NFIB phase separation, FRAP assays were performed. The results demonstrated that the mobility of NFIB droplets in WT cells was reduced after treatment with NMN, a SIRT family agonist, but no change in droplet mobility was observed in K65‐mutated cells (Figure 5G). This suggests that acetylation at K65 plays a pivotal role in reducing the mobility of NFIB droplets. Taken together, these findings indicate that K65 acetylation is a key determinant of NFIB condensate liquidity and dynamic behavior.

### 2.6 Liquid–Liquid Phase Separation of NFIB Is Required for Its Ferroptosis‐Suppressive Function

**FIGURE 6:**
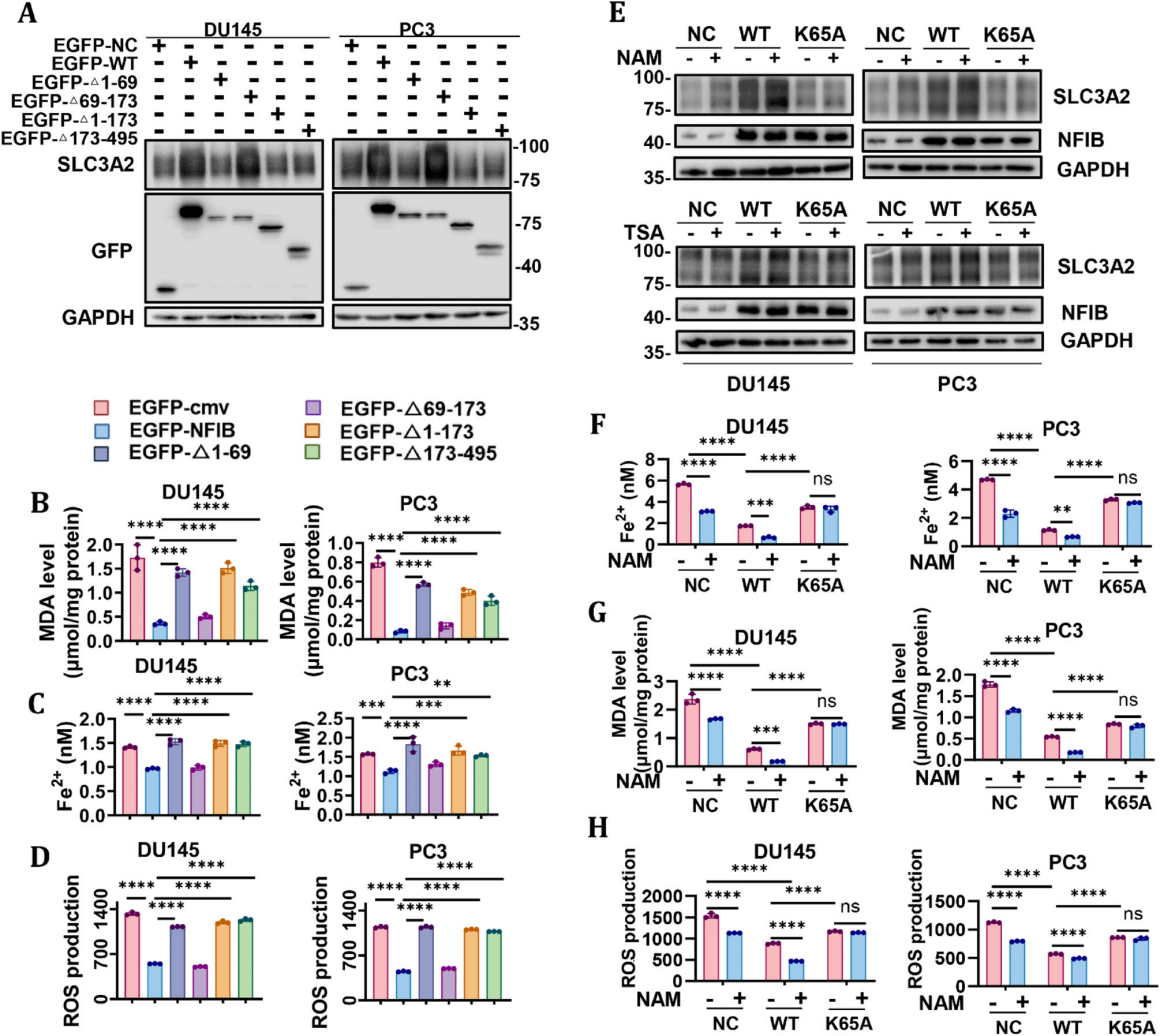
Acetylation at K65 regulates NFIB phase separation and affects downstream ferroptosis. (A) Protein expression of SLC3A2 in DU145‐sgNFIB and PC3‐sgNFIB cells transfected with NFIB phase‐separated segment deletion mutants following erastin treatment. (B)MDA level in DU145‐sgNFIB and PC3‐sgNFIB cells transfected with NFIB phase‐separated segment deletion mutants following erastin treatment. (C) Fe^2+^ measurement in DU145‐sgNFIB and PC3‐sgNFIB cells transfected with NFIB phase‐separated segment deletion mutants following erastin treatment. (D)ROS measurements in DU145‐sgNFIB and PC3‐sgNFIB cells transfected with NFIB phase‐separated segment deletion mutants following erastin treatment. (E) SLC3A2 expression in K65A mutants of DU145 and PC3 cells with NFIB knockout with or without NAM and TSA treatment. Vector plasmid NC and full‐length overexpression plasmid WT were used as controls. (F) Fe^2+^ measurement in K65A mutants of DU145 and PC3 cells with NFIB knockout with or without NAM treatment. (G) MDA level in K65A mutants of DU145 and PC3 cells with NFIB knockout with or without NAM treatment. (H) ROS measurements in K65A mutants of DU145 and PC3 cells with NFIB knockout with or without NAM treatment. The data were expressed as mean ± standard deviation from at least three independent experiments. Differences between groups were assessed using a two‐tailed unpaired Student's *t*‐test, or one‐way ANOVA test with Bonferroni's correction. *p ≤ 0.05, **p ≤ 0.01, ***p ≤ 0.001 and ****p ≤ 0.0001.

To determine whether the phase separation of NFIB directly influences ferroptosis, we examined SLC3A2 expression in NFIB‐knockout DU145 and PC3 cells reconstituted with each EGFP‐NFIB construct (Figure 6A). Cells expressing full‐length EGFP‐NFIB or EGFP‐NFIBΔ69‐173 maintained robust SLC3A2 protein levels, consistent with their preserved droplet formation and fluidity. In contrast, EGFP‐NFIBΔ1‐69, EGFP‐NFIBΔ1‐173, or EGFP‐NFIBΔ173‐495 led to a marked reduction in SLC3A2 expression. These results indicate that intact condensate formation mediated by both the C‐terminal and N‐terminal IDRs is required for efficient transcriptional activation of SLC3A2 by NFIB, further supporting the model that NFIB suppresses ferroptosis by phase‐separating in the nucleus to modulate key ferroptosis regulators. Then, we expressed each EGFP‐tagged construct in NFIB‐knockout CRPC cells and then treated them with erastin. Cells reconstituted with full‐length EGFP‐NFIB or the MH1‐domain deletion mutant EGFP‐NFIBΔ69‐173 displayed a marked reduction in erastin‐induced lipid peroxidation and iron accumulation, as evidenced by decreased MDA and ROS production, and lower Fe^2^^+^ levels (Figure 6B–D). In stark contrast, deletion of the N‐terminal IDR (EGFP‐NFIBΔ1‐69), the entire pre‐N‐terminal IDR and MH1 module (EGFP‐NFIBΔ1‐173), or the C‐terminal transcriptional modulation region (EGFP‐NFIBΔ173‐495) completely abrogated this protective effect, resulting in significantly heightened MDA and ROS levels and elevated Fe^2^^+^ content relative to EGFP‐NFIB–expressing cells (Figure 6B–D). These findings indicate that the capacity of NFIB to form liquid‐like condensates within the nucleus, driven by its C‐terminal IDR, and the maintenance of droplet fluidity, governed by its N‐terminal IDR, are both essential for its ferroptosis‐suppressive function.

Consistent with earlier observations, SLC3A2 expression was reduced upon mutation of K65 (Figure 5E) and was upregulated following NAM treatment in wild‐type cells (Figure 5F), suggesting that NFIB acetylation may influence ferroptosis through regulation of SLC3A2 expression. To clarify whether acetylation at K65 specifically affects ferroptosis, we examined the protein expression of SLC3A2 in CRPC cells with NFIB knockout at K65 following NAM treatment. Our results showed that NAM treatment led to an increase in SLC3A2 expression in WT cells, but did not affect the SLC3A2 levels in K65‐mutated cells (Figure 6E). Then, we evaluated the impact of K65 mutation on ferroptosis markers after NAM treatment. ROS level, MDA level and Fe^2+^ content were significantly increased in K65‐mutant cells compared to WT cells, and these changes could not be reversed by NAM treatment (Figure 6F–H). Taken together, these findings suggest that acetylation‐dependent regulation of NFIB phase separation functionally links NFIB condensate dynamics to ferroptosis suppression, highlighting a potentially targetable vulnerability in CRPC cells.

### 2.7 SIRT7 is an Important Protein That Regulates NFIB Deacetylation

**FIGURE 7:**
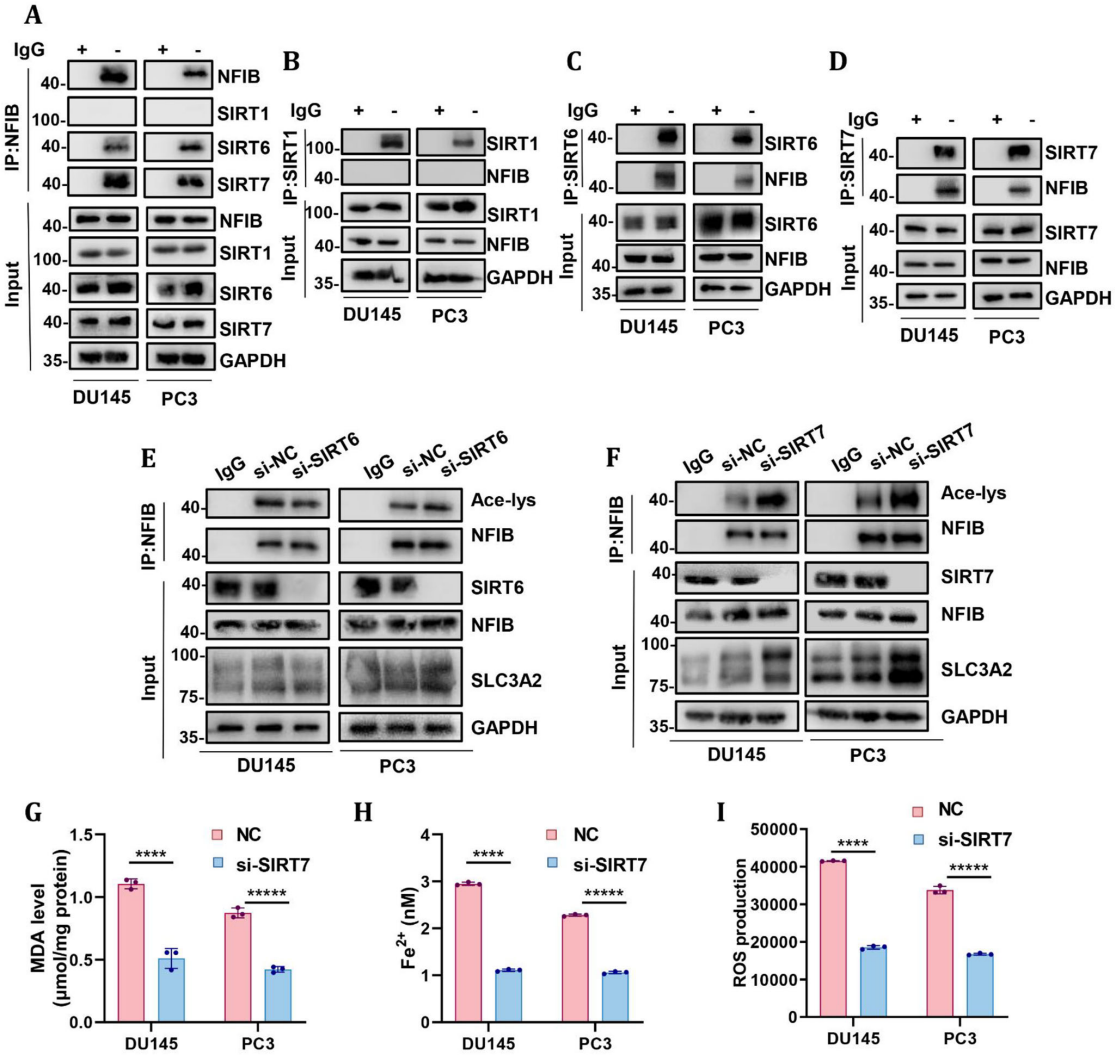
SIRT7 is an important protein that regulates NFIB deacetylation. (A–D) Co‐immunoprecipitation showed that NFIB interacted with SIRT6 and SIRT7 but not SIRT1 in DU145 and PC3 cells. (E) NFIB acetylation and SLC3A2 expression were measured in DU145 and PC3 cells with knockdown of SIRT6. (F) NFIB acetylation and SLC3A2 expression were measured in DU145 and PC3 cells with knockdown of SIRT7. (G) MDA level was measured in DU145 and PC3 cells with knockdown of SIRT7. (H) Fe^2^^+^ was measured in DU145 and PC3 cells with knockdown of SIRT7. (I) ROS production was measured in DU145 and PC3 cells with knockdown of SIRT7. All quantitative data are presented as mean ± SD from at least three independent biological replicates (n ≥ 3). Statistical significance was determined using a two‐tailed unpaired Student's t‐test. *p ≤ 0.05, **p ≤ 0.01, ***p ≤ 0.001, and ****p ≤ 0.0001.

Having established that acetylation at K65 within the N‐terminal IDR dynamically regulates NFIB phase separation and ferroptosis suppression, we next sought to identify the upstream deacetylase responsible for this modification. Because NFIB is predominantly localized in the nucleus, we focused our analysis on nuclear members of the SIRT family, including SIRT1, SIRT6, and SIRT7. Co‐IP assays revealed that NFIB interacts with both SIRT6 and SIRT7, but not with SIRT1(Figure 7A–D). To determine their functional roles, SIRT6 or SIRT7 was knocked down in CRPC cells. Interestingly, only knockdown of SIRT7 resulted in a marked increase in NFIB acetylation levels accompanied by upregulation of SLC3A2 protein expression, whereas SIRT6 knockdown had no such effect (Figure 7E,F). Furthermore, knockdown of SIRT7 led to reduced levels of MDA, ROS, and Fe^2^^+^ in both cell lines, indicating attenuated lipid peroxidation and iron accumulation, and consequently enhanced resistance to ferroptosis (Figure 7G–I). Together, these results identify SIRT7 as the primary nuclear deacetylase regulating NFIB acetylation and establish a SIRT7–NFIB–SLC3A2 axis controlling ferroptosis sensitivity in CRPC cells.

### 2.8 Targeting NFIB in Combination With Ferroptosis Inducers Inhibited Tumor Growth In Vivo

**FIGURE 8:**
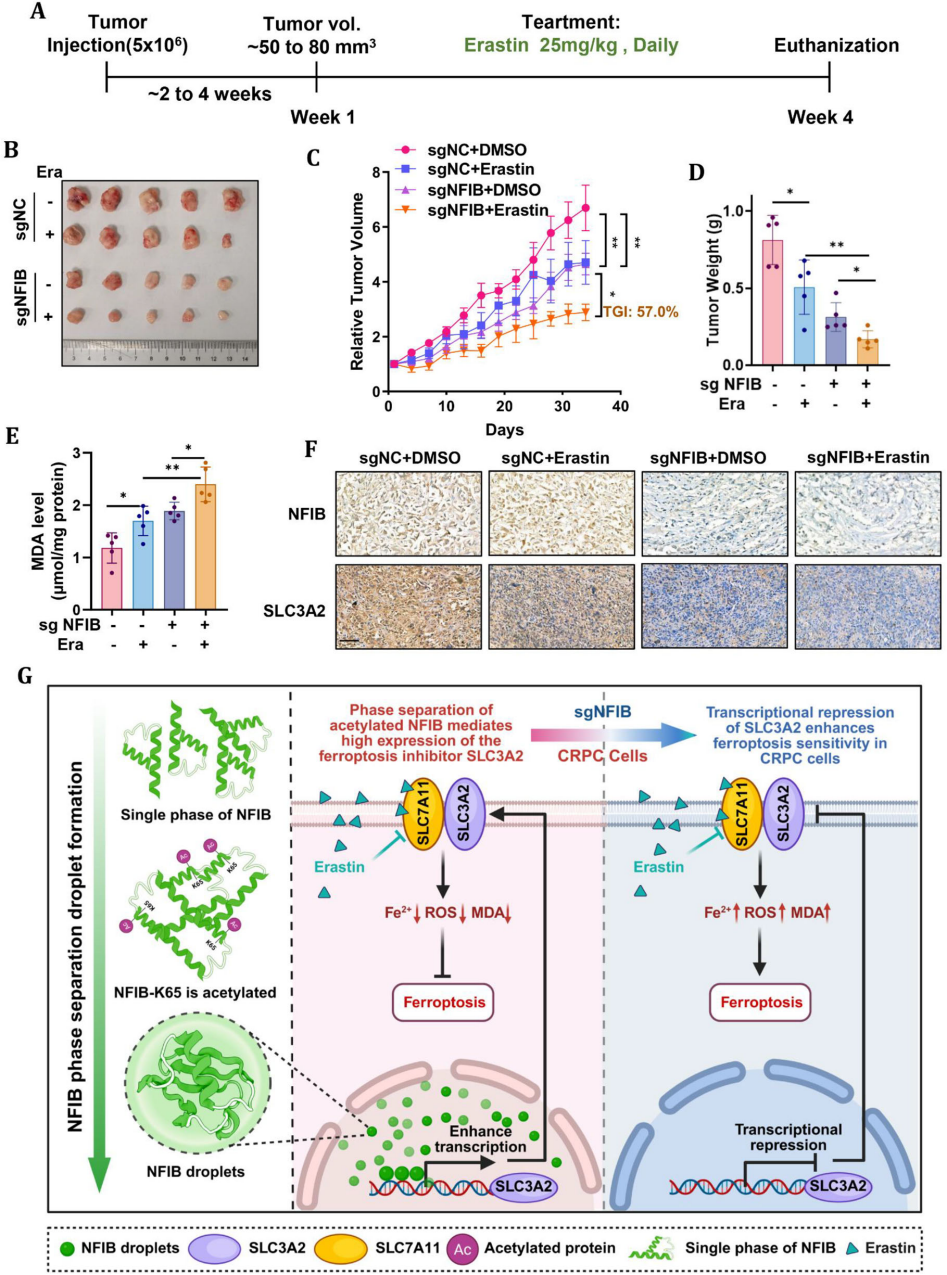
Targeting NFIB in combination with ferroptosis inducers inhibited tumor growth in vivo. (A) Schematic diagram of experimental design using erastin treatment. (B) Representative images of excised xenograft tumors from each treatment group (n = 5 mice per group). (C)The size of mice tumor xenograft was recorded in each group, and tumor growth curves were normalized to baseline volume (V/V0) to eliminate confounding from initial size variations. Here, V0 denotes the tumor volume measured on the first day of treatment (n = 5 per group). (D) After mice were sacrificed, tumor weights were measured. (E) MDA level of mice tumor in each group. (F) Tumor specimens were subjected to IHC staining for NFIB and SLC3A2. Scale bar, 50 µm. (G) Schematic summary of acetylated NFIB–mediated phase separation regulating ferroptosis via SLC3A2. Created with BioRender.com. The data were expressed as mean ± standard deviation. Differences between groups were assessed using one‐way ANOVA test with Bonferroni's correction. *p ≤ 0.05, **p ≤ 0.01, ***p ≤ 0.001, and ****p ≤ 0.0001.

At present, there is no effective treatment for CRPC. To assess the therapeutic potential of targeting NFIB in combination with ferroptosis inducers, we constructed subcutaneous tumor models in nude mice using DU145 cells stably knocked out for NFIB by CRISPR‐Cas9 technology. The mice were randomly divided into four treatment groups: sgNC+DMSO, sgNC+erastin, sgNFIB+DMSO, and sgNFIB+erastin. Tumor growth was monitored after the tumor volume reached 50–80 mm^3^, and drugs were administered accordingly. The erastin group received daily injections of erastin at 25 mg/kg, while the DMSO group was treated with the corresponding DMSO solution (Figure 8A). Tumor volumes and body weights were recorded every 3 days, and the data were graphed over time (Figure S4A).

The results showed that the combination of NFIB targeting and ferroptosis induction significantly inhibited tumor growth compared to the control groups (Figure 8B–D). Tumor growth curves were normalized to baseline volume (V/V0) to eliminate confounding from initial size variations (Figure 8C). Here, V0 denotes the tumor volume measured on the first day of treatment. Furthermore, tumor growth inhibition (TGI) was calculated as TGI (%) = [1 − (ΔV_treatment / ΔV_control)] × 100%, with the sgNC+DMSO group serving as the control. The calculated TGI values were as follows: sgNC+DMSO (100%), sgNC+erastin (29.7%), sgNFIB+DMSO (30.6%), and sgNFIB+erastin (57.0%). These results indicate that the sgNFIB+erastin group exhibited the most pronounced antitumor effect, showing the smallest absolute tumor volume along with the highest TGI value. Specifically, the sgNFIB+erastin group exhibited the most substantial reduction in tumor size. To further assess the impact of NFIB on metastatic progression and its contribution to ferroptosis‐based therapy, we established an experimental metastasis model by tail‐vein injection of sgNC or sgNFIB DU145 cells. Consistent with our previous report [22], sgNFIB DU145 xenografts exhibited a marked suppression of CRPC metastatic burden compared with sgNC controls. Importantly, NFIB depletion significantly enhanced the anti‐metastatic efficacy of erastin, as evidenced by reduced pulmonary metastatic load assessed by hematoxylin and eosin (HE) staining and quantitative enumeration of metastatic nodules. These results indicate that NFIB loss not only suppresses metastatic colonization, but also enhances the sensitivity of disseminated CRPC cells to erastin‐induced ferroptosis in vivo (Figure S4B,C).

Additionally, the MDA content in the sgNFIB+erastin group was the highest, indicating enhanced lipid peroxidation and increased ferroptotic damage within the tumors (Figure 8E). These findings suggest that the combination of NFIB inhibition and ferroptosis induction effectively promotes ferroptosis in the tumors, leading to a reduction in tumor growth. Immunohistochemical analysis of the tumor tissues confirmed the lower expression of NFIB in the sgNFIB group compared to controls, while the sgNFIB+erastin group exhibited the lowest levels of SLC3A2 expression among all groups (Figure 8F). This reduction in SLC3A2 expression further supports the activation of the ferroptosis pathway through NFIB targeting in combination with erastin treatment.

Importantly, no significant side effects were observed in the erastin‐treated animals, as evidenced by stable body weight and histological examination of the organs via HE staining (Figure S4D,E). We assessed various parameters to evaluate hepatotoxicity, including alanine aminotransferase (ALT), aspartate aminotransferase (AST), and lactate dehydrogenase (LDH). Additionally, we examined markers for nephrotoxicity, such as urea (UREA), uric acid (UA), and creatinine (CREA). In addition, myocardial and skeletal muscle toxicity were detected by creatine kinase isoenzyme MB (CK‐MB) and creatine kinase (CK). Our results showed no significant differences in any of these parameters between the sgNC+erastin, sgNFIB+DMSO, and sgNFIB+erastin group and the control group (Figure S4F–M). These results collectively demonstrate that the targeted inhibition of NFIB, in combination with ferroptosis inducers, is a promising therapeutic strategy for CRPC, without observable systemic toxicity.

## 3 Discussion

The treatment of CRPC is still very limited presently, and its refractory nature has become a difficult problem to be solved. The development of drug tolerance and the limitations of treatment regimens are two key issues [27, 28, 29]. In addition, CRPC is highly heterogeneous and can be subtyped according to androgen receptor (AR) status [30]. AR‐dependent CRPC is the most common subtype [31, 32], but some patients will gradually develop AR‐independent CRPC [33]. Although newer generation AR antagonists and AR pathway inhibitors have shown some efficacy, resistance remains a major challenge [34, 35, 36]. With the increase of ferroptosis related studies, the role of ferroptosis in cancer has been gradually revealed. In terms of drug resistance, many cancer cells are resistant to conventional chemotherapy drugs but sensitive to ferroptosis inducers [37, 38, 39]. In our study, we found that although CRPC cells are sensitive to ferroptosis inducers, there is still activation of the ferroptosis inhibitory pathway. By knockout of NFIB, which is highly expressed in CRPC, inhibition of the ferroptosis inhibitory pathway significantly increased the sensitivity of CPRC cells to ferroptosis inducers. This suggests that ferroptosis induction therapy targeting NFIB may become a potential strategy for the treatment of CRPC.

As a key member of the nuclear factor I family, NFIB plays an important role in tumorigenesis, progression and the regulation of related signaling pathways. The high expression of NFIB is associated with the progression and poor prognosis of a variety of tumors, including clear cell renal cell carcinoma and adenoid cystic carcinoma [20, 40]. However, little is known about the biological role of NFIB in prostate cancer. Our previous work demonstrated that high NFIB expression in CRPC is strongly correlated with metastatic dissemination [22]. This study provides another direction for the explanation of the refractory mechanism of CRPC from the perspective of ferroptosis. NFIB is expected to be a new negative regulator of ferroptosis and a potentially valuable molecular biomarker for CRPC. Note that in our previous in vivo study on NFIB and metastasis, knocking down NFIB (shNFIB) did not significantly inhibit tumor growth in mice, while in the present study, knocking out NFIB (sgNFIB) observed significant inhibition of tumor growth in mice, which may be due to the different construction of gene expression reduction system. With the CRISPR/Cas9 knockout system used in this study, NFIB was knocked out more thoroughly. In this study, we observed that sgNFIB itself reduced the tumor growth rate and volume. Consequently, when treatments were initiated after tumors had grown to a suitable size, the baseline tumor volumes varied across groups. To eliminate the potential confounding effect of these initial size differences, tumor growth curves were normalized to the baseline volume (V/V0). The normalized analysis revealed that the combination of NFIB targeting and erastin treatment resulted in the smallest absolute tumor volume. Moreover, the relative extent of tumor suppression was more pronounced in this combination group compared to the controls. Importantly, this cooperative effect was not limited to primary tumor growth, as NFIB depletion also markedly suppressed metastatic dissemination in a tail‐vein injection model and significantly potentiated the anti‐metastatic efficacy of erastin, as evidenced by reduced pulmonary metastatic burden. Together, these results indicate that NFIB targeting enhances ferroptosis‐based therapy by restraining both tumor growth and metastatic progression in CRPC. We acknowledge that more advanced patient‐derived models, including PDXs or organoid systems, would further enhance the clinical relevance of our findings. Future incorporation of these models will allow a more comprehensive assessment of NFIB function in heterogeneous CRPC contexts and provide additional translational validation of the NFIB–ferroptosis axis.

Biomolecular aggregates formed by LLPS play an important role in the spatiotemporal coordination of intracellular biological activities [41]. These biomolecular condensates are widely observed to be able to directly regulate key cellular processes related to cancer cell pathology, and LLPS dysregulation is increasingly recognized as a previously underappreciated driver of oncogenic activity that plays an important role in regulating gene expression [42, 43] and cell signaling [44, 45, 46]. We provide the first demonstration that NFIB forms liquid‐like condensates via LLPS in the nucleus, and that its N‐terminal and C‐terminal IDRs serve complementary roles in ferroptosis suppression. Specifically, the C‐terminal IDR (aa 173–495) drives condensate nucleation, creating localized microenvironments that facilitate stable binding of NFIB to the SLC3A2 promoter, while the N‐terminal IDR (aa 1–69) maintains droplet fluidity, enabling dynamic remodeling of these transcriptional hubs. Through this phase‐separation–mediated promoter recruitment, NFIB potently upregulates SLC3A2 transcription, thereby blocking iron accumulation and lipid peroxidation. Moreover, acetylation at K65 within the N‐terminal IDR fine‐tunes condensate liquidity, enhancing transcriptional control of NFIB over SLC3A2 and reinforcing ferroptosis resistance in CRPC. These findings uncover a novel mechanism wherein NFIB leverages LLPS and Post‐translational modifications (PTM) regulated droplet dynamics to orchestrate the expression of a key ferroptosis inhibitor, significantly expanding our understanding of transcriptional regulation via phase‐separated condensates (Figure 8G). However, our inability to purify full‐length and truncated NFIB protein limits in vitro biophysical characterization, and the molecular basis for C‐terminal–driven nuclear export upon droplet failure remains to be elucidated.

PTMs are of great significance in the study of cancer mechanisms, the common forms of post‐translational modifications include phosphorylation, ubiquitination, methylation and acetylation [47]. Acetylation plays an important role in the occurrence and development of cancer [48]. Our study explored the effects of NFIB acetylation on phase separation and ferroptosis in CRPC, and clarified that the non‐histone acetylation of NFIB is regulated by SIRT family rather than HDAC family, and SIRT7 is mainly responsible for the deacetylation of NFIB, which broadened the research boundary of NFIB acetylation. Future studies should map the complete enzymatic network regulating NFIB acetylation and determine how these PTMs integrate with other modifications to orchestrate NFIB phase‐separation–dependent gene regulatory program.

Although transcription factors such as NFIB are traditionally regarded as difficult drug targets, recent progress in nucleic‐acid–based approaches and transcription factor–degradation strategies has enabled functional suppression of NFIB at the expression or protein level, despite the lack of conventional small‐molecule inhibitors [49, 50]. More importantly, the SIRT7–NFIB–SLC3A2 axis defines a drug‐actionable regulatory pathway, in which SIRT7 governs NFIB acetylation and phase‐separation dynamics to control SLC3A2 transcription (Figure 7). Pharmacologic sirtuin modulators such as resveratrol, which have been reported to influence nuclear sirtuin activity in cellular contexts [51], may attenuate NFIB‐driven ferroptosis resistance by destabilizing NFIB phase separation. At the effector level, ferroptosis inducers such as erastin directly inhibit System Xc^−^–mediated cystine uptake. Therefore, combining NFIB‐directed strategies, either directly or via SIRT7 modulation, with erastin represents a rational vertical combination strategy to lower the ferroptotic threshold and enhance anti‐tumor efficacy in CRPC.

In summary, our study reveals that the ability of NFIB to form and dynamically modulate nuclear condensates via its N‐ and C‐terminal IDRs underpins a previously unrecognized transcriptional mechanism controlling ferroptosis in CRPC. By phase‐separating on the SLC3A2 promoter and fine‐tuning condensate liquidity through K65 acetylation, NFIB orchestrates the expression of a key anti‐ferroptosis factor, thereby driving CRPC resistance to cell death. These insights establish NFIB as both a mechanistic nexus linking LLPS and ferroptosis regulation and a compelling therapeutic target. Targeting condensate dynamics of NFIB, either by disrupting its phase separation or modulating its PTM network, holds promise for sensitizing refractory CRPC to ferroptosis‐based treatment strategies and warrants further translational development.

## 4 Materials and Methods

### 4.1 Cell Lines and Cell Culture

The human prostate cancer cell lines VCaP (Cat# CRL‐2876; RRID: CVCL_2235) LNCaP (Cat# CRL‐1740; RRID: CVCL 0395), DU145 (Cat# HTB‐81; RRID: CVCL 0105) and PC3 (Cat# CRL‐1435; RRID: CVCL 0035), were obtained from the American Type Culture Collection (ATCC). Mycoplasma was detected negative in all cell lines. LNCaP cells were cultured in in RPMI‐1640 (Gibco, USA) supplemented with 10% FBS (Gibco, USA). VCaP cells were cultured in in DMEM (Gibco, USA) supplemented with 10% FBS (Gibco, USA). DU145 and PC3 CRPC cells were respectively cultured in MEM (Gibco, USA) and F12 (Gibco, USA) media supplemented with 10% FBS. All cells were maintained at 37°C in a humidified incubator with 5% CO2.

### 4.2 Plasmids, siRNA and Cell Transfection

NFIB was knocked out using the LentiCRISPRv2 vector and stably knocked out using CRISPR Cas9 gene editing technology in our laboratory. The two target sequences for NFIB knockout were CACCGACTGCCAAAGATATTCGCC and AAACGGCGAATATCTTTG‐CGCAGTC. After CRISPR/Cas9 transfection and puromycin selection, single‐cell–derived clones were isolated by limiting dilution and individually expanded. Knockout efficiency was rigorously validated at the protein level by Western blot analysis to confirm the complete absence of NFIB expression, indicating successful biallelic disruption. Two independent single‐cell clones exhibiting complete NFIB knockout were selected for validation experiments to exclude clonal variability. A representative single clone with confirmed complete NFIB knockout was selected and used consistently for all subsequent in vitro and in vivo functional experiments. The pEZX‐PG04.1‐Fluc reporter was synthesized by GeneCopoeia (China). The phase separation segment deletion plasmid was constructed in our laboratory using the pEGFP‐C1 vector. The Precever‐LV201 vector and its corresponding point mutation were constructed by GeneCopoeia (China). The siRNAs used in this study were synthesized by HanYi Biosciences Inc (Guangzhou, China), and the sequences of the siRNAs are listed in Table S3. TurboFect Transfection Reagent (Thermo Fisher Scientific, USA) was used for transfection, following the manufacturer's instructions. The efficiency of transfection was confirmed using quantitative real‐time PCR or western blotting assays.

### 4.3 Gene‐Set Enrichment Analysis

Gene‐Set Enrichment Analysis (GSEA) analysis of Ferroptosis pathway using database FerrDb (http://www.zhounan.org/ferrdb/current/) provides the analysis of the plate were analyzed [52]. The analysis is performed using GSEApy, with both Enrichr's over‐representation and Subramanian et al.'s GSEA methods available [53, 54].

### 4.4 Luciferase Reporter Assay

The pEZX‐PG04.1‐SLC3A2‐Fluc reporter and the mutant pEZX‐PG04.1‐SLC3A2‐Fluc reporter with NFIB binding sites mutation were constructed to detect the alteration of promoter activity. The luciferase reporter assay was performed using the Dual Luminescence Assay Kit (GeneCopoeia, China) according to the manufacturer's instructions.

### 4.5 RNA Extraction and Quantitative Real‐Time PCR

RNA extraction using Goonie (China) and quantitative real‐time qPCR were performed following the protocol outlined in our previous study [55]. The sequences of the primers used are listed in Table S4.

### 4.6 Immunohistochemical (IHC) Staining and Western Blotting

Western blotting and IHC assays were conducted as previously described [22]. The primary antibodies used in Western blotting included anti‐NFIB (1:1000, Abcam), anti‐SLC3A2 (1:1000, CST), anti‐SLC7A11 (1:1000, CST), anti‐GAPDH (1:5000, proteintech), anti‐Ace‐lys (1:1000, CST). Raw data of the western blot are plotted in the supplementary material Original Western blot. The primary antibodies used in IHC included anti‐NFIB (1:250, HUABIO), anti‐ SLC3A2 (1:250, HUABIO).

### 4.7 ROS Measurement

2 × 10^5^ cells were grown in 6‐well plates overnight at 37°C. The next day, cells were treated with 1 µM erastin (Selleck, USA) for 6 h followed by the addition of 10 µM DCFH‐DA (Beyotime, Shanghai, China), for 20 min. For cells transfected with EGFP plasmids, the cells were incubated with 10 µM DHE (sorlabio, Beijing, China) instead of DCFH‐DA for 40 min. Cells were washed with PBS and harvested with Trypsin/EDTA (0.25%). Cells were subjected to flow cytometry to measure the levels of cellular ROS.

### 4.8 Iron Assay and Malondialdehyde Assay

1 × 10^6^ cells were grown in 10 cm dish overnight at 37°C. The next day, cells were treated with 1 µM erastin for 12 h. The respective lysates of the Iron assay (Abcam, UK) and Lipid Peroxidation MDA Assay Kit (Beyotime, Shanghai, China) were added and assayed according to the instructions. OD values were read using a microplate reader with Malondialdehyde (MDA) at a wavelength of 532 nm and Iron (Fe^2+^) at a wavelength of 593 nm.

### 4.9 Cell Viability and Cell Death Assays

For viability assays, cells were seeded in 96‐well plates and treated with drugs for the indicated durations the following day. The medium containing drugs was then aspirated and replaced with fresh medium supplemented with 10% Cell Counting Kit‐8 (CCK‐8) reagent (APExBIO, Houston, USA). Following incubation at 37°C for 2 h, absorbance was measured at 450 nm using a microplate reader (BioTek, USA), with background subtraction using appropriate blank wells (medium only). To quantify cell death, cells were seeded in 12‐well plates and treated with drugs for the indicated time periods the following day. Following treatment, cells were harvested by trypsinization, centrifuged to obtain cell pellets, and resuspended in phosphate‐buffered saline (PBS). The cell suspension (100 µL) was mixed with 0.02% trypan blue solution (200 µL) and incubated for 3 min at room temperature. The proportion of dead cells (trypan blue‐positive) was determined using a Countess II FL automated cell counter (Model JSY‐SC‐031N, BodBoge, Shenzhen, China). The percentage of cell death was calculated as the ratio of trypan blue‐positive cells to the total cell count.

### 4.10 GSH Measurement Assays

Cells were seeded in 6‐well plates with appropriate treatment. Following treatment, cells were harvested by trypsinization, washed twice with ice‐cold PBS, and resuspended in PBS to prepare cell suspensions. Intracellular reduced glutathione (GSH) levels were measured using the Micro Reduced Glutathione (GSH) Content Assay Kit (Solarbio, Beijing, China) according to the manufacturer's instructions. Briefly, cell suspensions were lysed and deproteinized using the provided extraction buffer, and the resulting supernatants were subjected to the DTNB colorimetric reaction. Absorbance was measured at 412 nm using a microplate reader. GSH concentration was calculated based on a concurrently run standard curve and normalized to the total protein content of the supernatant, determined by a BCA or Bradford assay, and expressed as µmol/mg protein.

### 4.11 Fluorescence Recovery After Photobleaching Assay

The fluorescence recovery after photobleaching (FRAP) experiments were performed using a confocal microscope (ZEISS LSM) equipped with 63 oil immersion objectives. EGFP‐NFIB bodies were bleached until the fluorescence intensity was reduced to 30% of the initial value. Images were taken every 3 s while the bleaching was being done at 80% laser power (488 nm). Following a previously described method [56], photobleaching during image acquisition was controlled by determining whether the fluorescence values of the unbleached spots were stable. Every data point shows the fluorescence intensity with standard and mean deviation of three bleached particles.

### 4.12 Immunofluorescence Staining of FFPE Sections

Paraffin‐embedded tissue blocks were sectioned at 4–5 µm. Sections were baked at 60°C for 30–60 min, deparaffinized using an eco‐friendly clearing agent, and rehydrated through a graded ethanol series. Heat‐induced antigen retrieval was performed in citrate buffer using a high‐temperature, high‐pressure method for 3 min, followed by natural cooling to room temperature. Sections were rinsed with PBS and permeabilized with 0.2% Triton X‐100 for 30 min. After washing, sections were incubated with 0.1 M glycine for 1 h to quench reactive aldehydes, then blocked with blocking solution (5% BSA, 0.1% Tween‐20 in PBS) for 120 min at room temperature. Primary antibodies were applied and incubated overnight at 4°C. The next day, sections were washed three times in PBS (5 min each) and incubated with fluorophore‐conjugated secondary antibodies for 1 h at room temperature in the dark. Nuclei were counterstained with DAPI for 10 min. Sections were washed with PBS and mounted with an antifade mounting medium. Images were acquired on a Leica confocal microscope. All groups were imaged using identical laser power, exposure, and detector gain settings.

### 4.13 Transmission Electron Microscopy of Cells

Cells were harvested by trypsinization and pelleted (800–1200 × g, 5 min). Cell pellets were gently washed with PBS and fixed overnight at 4°C in pre‐chilled 2.5% glutaraldehyde prepared in 0.1 M phosphate buffer (pH 7.2–7.4). Samples were washed three times with the same buffer (10 min each) and then processed by the electron microscopy core facility. Mitochondrial ultrastructure was examined by transmission electron microscopy.

### 4.14 Animal Models

The animal experiments in this study were approved by the Animal Ethics Committee of the Fifth Affiliated Hospital of Sun Yat‐sen University. For prostate cancer xenograft models, a total of 5 × 10^6^ cells were injected subcutaneously into 4‐week‐old male BALB/c nude mice. Tumor growth was monitored by calipers, and tumor volume was calculated using the formula: V = 1/2 × larger diameter × (smaller diameter)^2^, and mice weight were recorded approximately every 3 days. At the end of the studies, the mice were sacrificed, and tumors were removed for further study. In addition, we constructed metastatic tumor models to evaluate the effect of NFIB on tumor metastasis. DU145‐sgNC/sgNFIB cells (1 × 10^6^ cells per mouse) were injected into the circulatory system of male Balb/c nude mice (6–8 weeks old, weighted 22–24 g). After approximately 8 weeks of feeding, the mice were sacrificed to observe the metastasis conditions in lung.

### 4.15 Statistical Analysis

The data were expressed as mean ± standard deviation from at least three independent experiments unless otherwise specified. Differences between groups were assessed using a two‐tailed unpaired Student's t‐test, non‐parametric test (Mann‐Whitney test), or one‐way ANOVA test with Bonferroni's correction. All analyses were conducted using GraphPad Prism 9.0 (La Jolla, CA, USA), and a two‐tailed value of *p ≤ 0.05, **p ≤ 0.01, ***p ≤ 0.001, and ****p ≤ 0.0001 were considered statistically significant.

## Author Contributions

Guanmin Jiang, Hao Liu, and Xiaohui Chen designed the outline of this manuscript. Qiunuo Li, Danyang Chen, Yongzhen Xia, and Yili Long were responsible for writing the initial manuscript and depicting all the figures and tables. Nan Huang, Rongna Li, Mengting Shi, Mairehaba Kadier, and Huafeng Luo provided technical assistance, helped to collect clinical samples, reviewed the manuscript and amended the reference. Guanmin Jiang, Hao Liu, and Xiaohui Chen revised the manuscript. All authors have read and approved the article.

## Ethics Statement

The obtainment of clinical samples and related experiments were approved by the Medical Ethics Committee of Fifth Affiliated Hospital of Sun Yat‐sen University [Approval No. 2022‐K204‐1]. Written informed consent was obtained from all participants. The animal experimental protocols of this study were reviewed and approved by the Fifth Affiliated Hospital of Sun Yat‐sen University [Approval No. 00417].

## Consent for Publication

The authors confirmed that we are consent for publishing the manuscript.

## Conflicts of Interest

The authors declare no conflict of interest.

## Supporting information




**Supporting File 1**: advs74637‐sup‐0001‐SuppMat.pdf.

**Supporting File 2**: advs74637‐sup‐0002‐TableS1‐S4.docx.

**Supporting File 3**: advs74637‐sup‐0003‐DataFile.zip.

## Data Availability

The accession numbers of the ChIP‐seq data for NFIB analyzed in this study is GEO at GSE219265. The data that support the findings of this study are available from the corresponding author upon reasonable request.
