# Supplementary material for: Phase Separation of NFIB Suppresses SLC3A2‐Mediated Ferroptosis in Castration‐Resistant Prostate Cancer
Source: Adv Sci (Weinh). 2026 Mar 9;13(26):e15340. doi: 10.1002/advs.202515340 (PMC13159144; doi:10.1002/advs.202515340)
Supplement: Supplementary file 1 — Supporting File 1: advs74637‐sup‐0001‐SuppMat.pdf. [file ADVS-13-e15340-s003.pdf]

Supplemental Fig.1

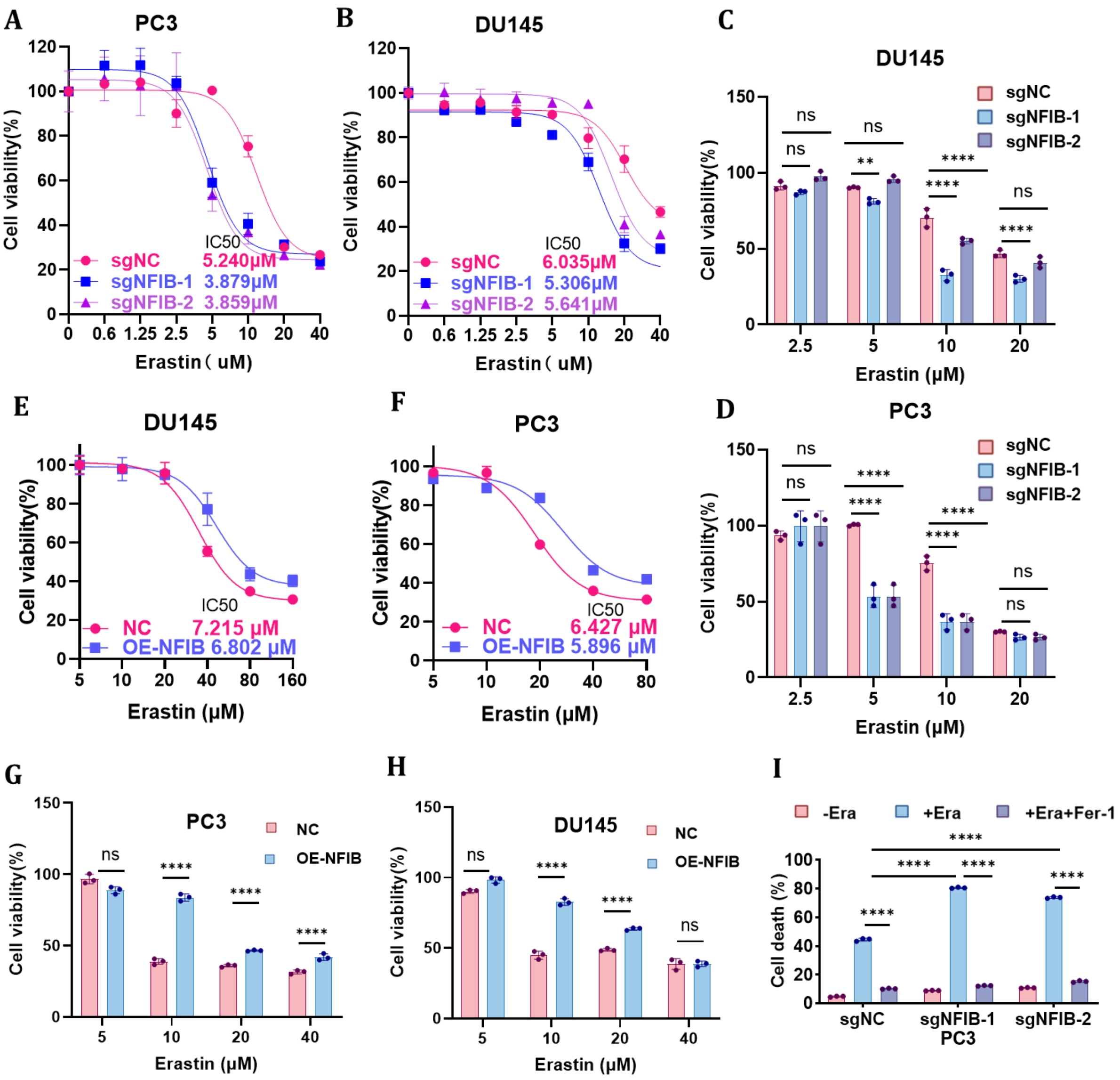

Figure S1. NFIB modulates cellular sensitivity to erastin in a dose-dependent manner.

(A, B) Cell viability of CRPC cells (DU145, PC3) with knockout of NFIB following different does of Erastin.

(C, D) NFIB knockout enhances ferroptosis sensitivity. Cell viability of DU145 and PC3 cells with stable NFIB knockout or corresponding control cells following treatment with increasing concentrations of erastin (2.5–20 μM) for 24 h, as assessed by CCK-8 assays.

(E, F) Cell viability of CRPC cells (DU145, PC3) with overexpression of NFIB following different does of Erastin.

(G, H) NFIB overexpression confers resistance. Cell viability of DU145 and PC3 cells with

NFIB overexpression or vector controls treated with increasing concentrations of erastin (5–40 μM) for 24 h, measured by CCK-8 assays.

(I) Cell death asssay in control or NFIB stable knockout PC3 cells treated with erastin in the presence or absence of fer-1.

Data are presented as mean ± SD from three independent experiments (n = 3). Statistical significance was determined using two-tailed unpaired Student's t-test between NFIB-manipulated cells and their respective controls at each erastin concentration. *P ⩽ 0.05, **P ⩽ 0.01, ***P ⩽ 0.001, ****P ⩽ 0.0001.

Supplemental Fig.2

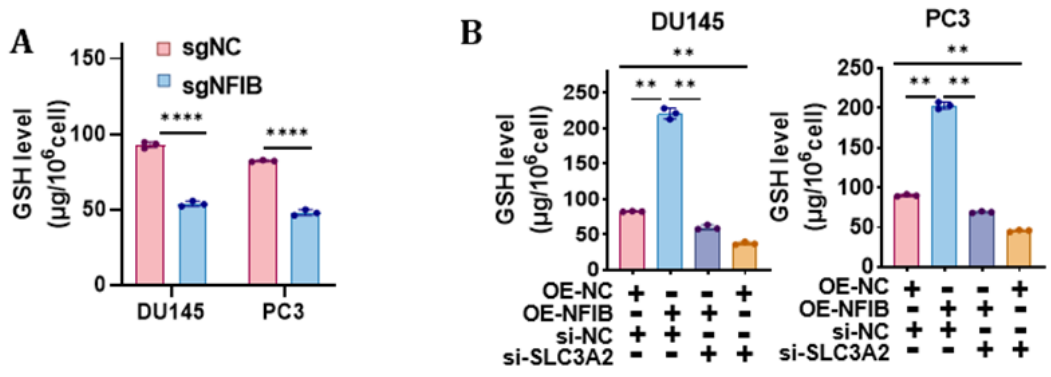

Figure S2. GSH levels in CRPC cells treated with different knockdown or overexpression treatments.

(A) GSH level of control or NFIB stable knockout DU145 and PC3 cells following Erastin treatment.

(B) GSH levels in DU145 and PC3 cells overexpressing NFIB or control vector, combined with knockdown of SLC3A2 or control.

Data are presented as mean ± SD from three independent experiments (n = 3). Statistical significance was determined using a two-tailed unpaired Student's t-test for comparisons between two groups or one-way ANOVA followed by Bonferroni post hoc correction for multiple-group comparisons. *P ⩽ 0.05, **P ⩽ 0.01, ***P ⩽ 0.001, ****P ⩽ 0.0001.

Supplemental Fig.3

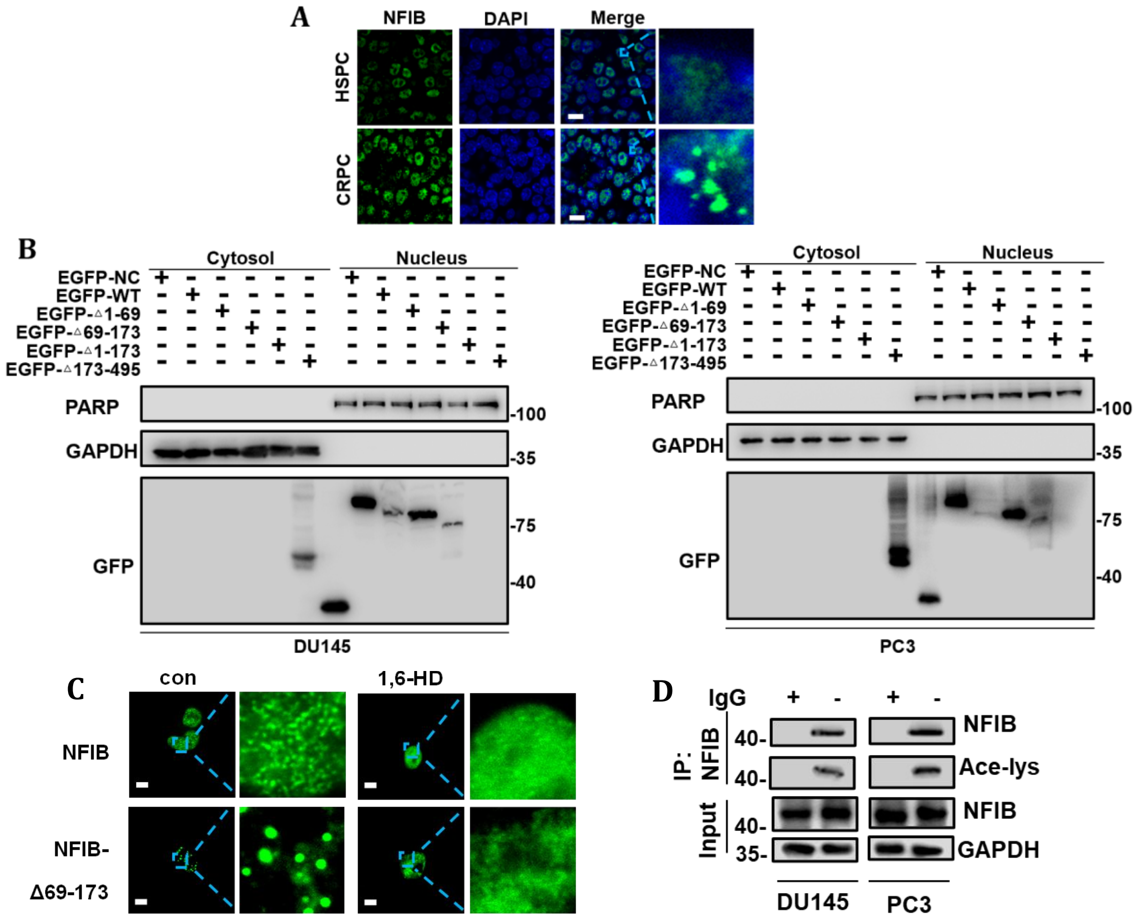

Figure S3. Phase separation ability of NFIB and its subcellular localization, and acetylation assays.

(A) Representative images of NFIB in mice tumor tissue. Scale bar, 20 μm.

(B) Nuclear-cytoplasmic fractionation of NFIB mutants in DU145 and PC3 with NFIB knockout.

(C)Representative images treated with or without 1, 6-hexanediol in PC3 cells. Scale bar, 10 μm.

(D) Immunoprecipitation assay showed that NFIB was acetylated in DU145 and PC3 cells.

Supplemental Fig.4

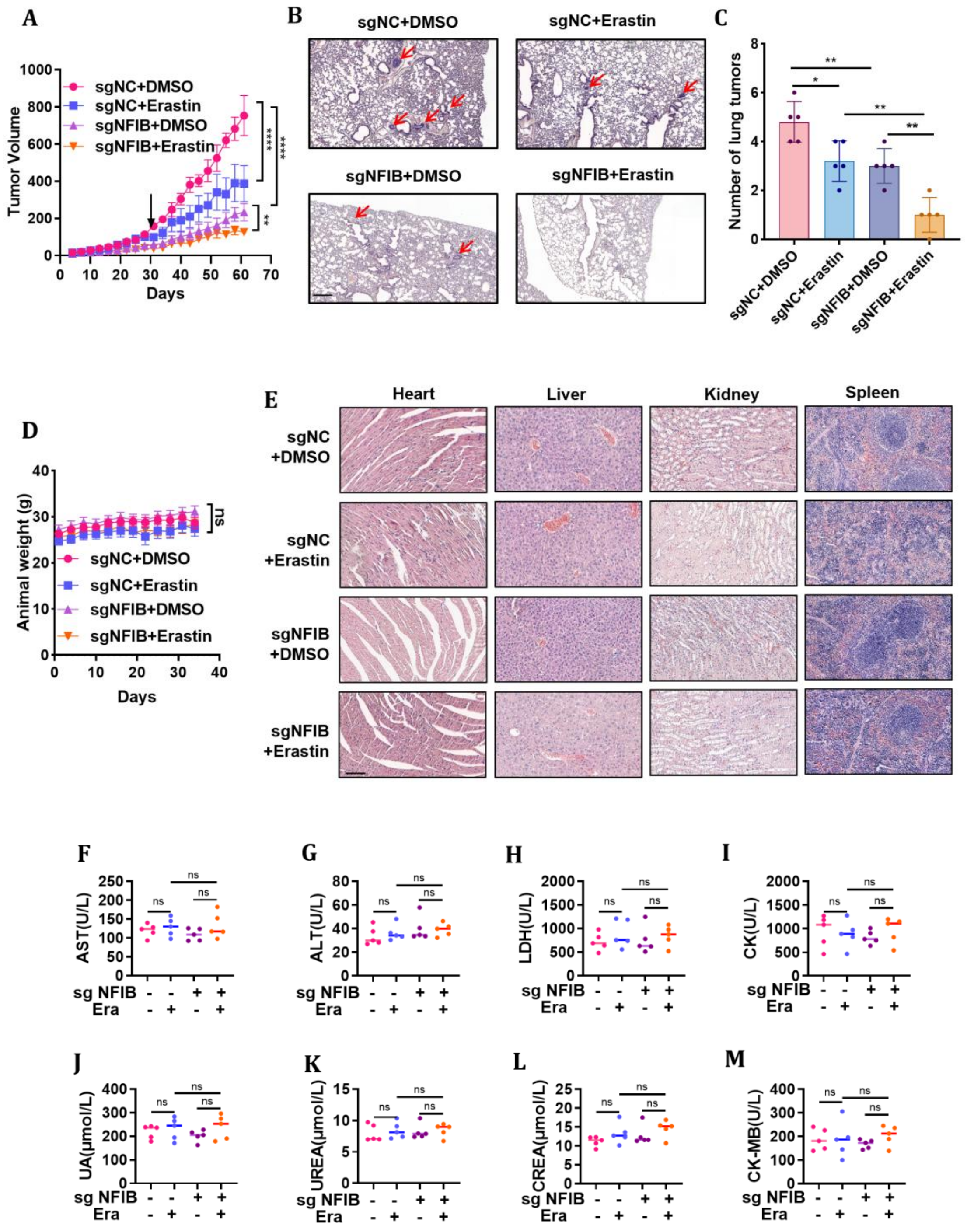

Figure S4. In vivo experiments confirmed that intervention NIFB in combination with ferroptosis promoter had good safety.

(A) The size of mice tumor xenograft was recorded in each group and the tumor volume growth curve was plotted.

(B) Representative images of metastatic lung tumors are shown and the locations of metastatic tumors pointed out with red arrows. Scale bar, 50 μm.

(C) The number of lung metastases in each group of mice (n = 5 per group).

(D) Animal weights were measured every three days for each group.

(E) Photographs of H&E staining of major organs from each group. Scale bar, 100 μm.

(F-M) Serum biochemical analysis demonstrated no drug-induced cardiotoxicity, hepatotoxicity, or nephrotoxicity in treated mice.

Data are presented as mean ± SD from five independent experiments (n = 5). Statistical significance was determined using a two-tailed unpaired Student's t-test for comparisons between two groups or one-way ANOVA followed by Bonferroni post hoc correction for multiple-group comparisons. $*P \leqslant 0.05$, $**P \leqslant 0.01$, $***P \leqslant 0.001$, $****P \leqslant 0.0001$.
