# Supplementary material for: Phase Separation of NFIB Suppresses SLC3A2‐Mediated Ferroptosis in Castration‐Resistant Prostate Cancer
Source: Adv Sci (Weinh). 2026 Mar 9;13(26):e15340. doi: 10.1002/advs.202515340 (PMC13159144; doi:10.1002/advs.202515340)
Supplement: Supplementary file 2 — Supporting File 2: advs74637‐sup‐0002‐TableS1‐S4.docx. [file ADVS-13-e15340-s002.docx]

**Table S1 FPKM of differently ferroptosis pathway regulatory genes expressed in prostate cells（LNCap,DU145 and PC3)**

| gene_id | LNCAP-1 | LNCAP-2 | LNCAP-3 | DU145-1 | DU145-2 | DU145-3 | PC3-1 | PC3-2 | PC3-3 |
| --- | --- | --- | --- | --- | --- | --- | --- | --- | --- |
| ACSL1 | 38.43821796 | 37.76856 | 36.88573 | 2.582489 | 2.829776 | 2.983165 | 14.3426 | 16.08552 | 15.17999 |
| ACSL3 | 64.22601925 | 65.15574 | 68.91715 | 10.81952 | 11.99202 | 12.48681 | 23.3047 | 23.97558 | 23.11277 |
| ACSL4 | 0.687926446 | 0.782248 | 0.651828 | 8.655754 | 11.39818 | 11.75159 | 15.91628 | 17.04087 | 17.23867 |
| ACSL5 | 0.335633629 | 0.352295 | 0.286219 | 0.690536 | 0.864637 | 1.098496 | 1.342596 | 1.348642 | 1.509469 |
| ACSL6 | 0.284394366 | 0.166876 | 0.232419 | 0.068662 | 0.122774 | 0.082803 | 0 | 0.008203 | 0.008011 |
| ALOX15 | 61.71842541 | 64.38981 | 60.63937 | 0 | 0.019714 | 0 | 0.082078 | 0 | 0.720366 |
| ATG5 | 13.39808819 | 15.0751 | 13.53275 | 6.120766 | 6.779723 | 6.482702 | 12.96193 | 13.2629 | 12.00879 |
| ATG7 | 3.970914394 | 4.053452 | 3.731653 | 3.936911 | 4.165173 | 4.164382 | 5.826118 | 6.008267 | 6.558697 |
| CYBB | 0 | 0 | 0 | 0.036658 | 0.110611 | 0.180849 | 0.153511 | 0.11825 | 0.179641 |
| FTH1 | 304.766306 | 302.387 | 310.8219 | 77.90086 | 71.14634 | 67.34897 | 97.63777 | 105.2467 | 103.42 |
| GPX4 | 98.880435 | 99.48791 | 96.39785 | 229.4553 | 226.6687 | 223.8208 | 329.0322 | 313.7965 | 319.2802 |
| GSS | 58.35423825 | 60.34922 | 58.71918 | 45.80853 | 43.29442 | 42.73224 | 26.79894 | 30.59125 | 28.76622 |
| LPCAT3 | 26.12993456 | 27.60657 | 26.5627 | 18.664 | 17.31626 | 16.83337 | 10.70256 | 12.4532 | 11.22629 |
| MAP1LC3B | 27.22214257 | 25.05295 | 25.88984 | 56.4753 | 52.44261 | 51.96936 | 56.30053 | 52.66731 | 48.55325 |
| NCOA4 | 59.46710354 | 57.07172 | 56.23754 | 40.19881 | 48.2456 | 49.35661 | 34.95355 | 32.61991 | 34.19126 |
| NFIB | 0.690081902 | 0.617028 | 0.559786 | 2.924877 | 3.329139 | 3.821146 | 3.833459 | 3.57498 | 3.78681 |
| PCBP2 | 141.7141243 | 147.993 | 142.6291 | 181.2995 | 171.6077 | 172.4346 | 105.8254 | 107.7962 | 103.0374 |
| PRNP | 45.1468818 | 44.50449 | 46.26016 | 49.25436 | 54.05195 | 52.28426 | 199.6836 | 194.543 | 197.1024 |
| SAT1 | 84.46291017 | 81.58836 | 87.92728 | 33.0294 | 31.48119 | 30.44629 | 61.64025 | 61.12466 | 62.96854 |
| SAT2 | 37.53531033 | 39.40783 | 39.16989 | 27.50606 | 21.18641 | 20.22889 | 23.9794 | 22.53963 | 21.74379 |
| SLC11A2 | 12.14740158 | 11.60677 | 11.90215 | 7.946819 | 9.345503 | 10.00802 | 10.47744 | 11.56767 | 11.39635 |
| SLC39A14 | 23.77950248 | 22.87467 | 23.99312 | 22.4585 | 28.1944 | 28.26415 | 29.29485 | 28.55422 | 30.40298 |
| SLC3A2 | 1.080257695 | 1.391024 | 1.520199 | 7.707367 | 7.642726 | 8.538778 | 5.436422 | 6.226995 | 6.397379 |
| SLC40A1 | 0.030277261 | 0.091368 | 0.065984 | 0.058479 | 0.088228 | 0.129826 | 0.397948 | 0.534484 | 0.568035 |
| SLC7A11 | 7.981062944 | 7.475251 | 9.000342 | 32.60365 | 35.47487 | 35.64242 | 24.64616 | 21.05265 | 22.30449 |
| STEAP3 | 3.901726424 | 4.395739 | 4.555215 | 12.9188 | 13.89254 | 14.03219 | 15.28322 | 15.39606 | 16.26807 |
| TFRC | 59.94228656 | 58.33691 | 56.91456 | 48.85132 | 64.84211 | 70.97805 | 146.3855 | 139.9533 | 144.4085 |
| TP53 | 59.7744523 | 59.97287 | 57.09646 | 31.75646 | 34.01382 | 31.57787 | 1.726195 | 1.400618 | 2.804949 |
| VDAC2 | 79.83965034 | 80.51979 | 74.93268 | 184.7816 | 164.521 | 162.7041 | 187.8415 | 181.3223 | 177.3163 |
| VDAC3 | 88.6480021 | 89.23703 | 87.29468 | 107.5387 | 98.70112 | 99.40211 | 50.13275 | 43.53997 | 48.40213 |

**Table S2 Primers for ChIP-qPCR assay**

| Gene | Sequence |
| --- | --- |
| SLC3A2-P1 | forward: 5’-CACAGAAGGCATAGTGCGTG-3’ |
|  | reverse: 5’-CTCGTGGTAAGTGGCTTCGT-3’ |
| SLC3A2-P3 | forward: 5’-GCTACAACACAGAAGGCATAGTG-3’ |
|  | reverse: 5’-GTGGTAAGTGGCTTCGTGG-3’ |

**Table S3 Sequence for siRNAs**

| Name | Sequence (5’-3’) |
| --- | --- |
| si SIRT6-1 | AGCGGAAGGUGUGGGAACUTT   AGUUCCCACACCUUCCGCUTT |
| si SIRT6-2 | GAAUGUGCCAAGUGUAAGATT   UCUUACACUUGGCACAUUCTT |
| si SIRT6-3 | GGAAGAAUGUGCCAAGUGUTT   ACACUUGGCACAUUCUUCCTT |
| si SIRT7-1 | UAGCCAUUUGUCCUUGAGGAATT UUCCUCAAGGACAAAUGGCUATT |
| si SIRT7-2 | GAACGGAACUCGGGUUAUUTT   AAUAACCCGAGUUCCGUUCTT |
| si SIRT7-3 | GAACGGAACUCGGGUUAUUTT   AAUAACCCGAGUUCCGUUCTT |

**Table S4 Primers for RT-qPCR assay**

| Gene | Sequence |
| --- | --- |
| GAPDH | forward: 5’- GTCTCCTCTGACTTCAACAGCG -3’ |
|  | reverse: 5’- ACCACCCTGTTGCTGTAGCCAA -3’ |
| NFIB | forward: 5’- AGAGATCAAGATATGTCTTC -3’ |
|  | reverse: 5’- CTGGCTGGTTTGTGGACTGGA -3’ |
| SLC3A2 | forward: 5’- CCAGAAGGATGATGTCGCTCAG -3’ |
|  | reverse: 5’- GAGTAAGGTCCAGAATGACACGG -3’ |
| GPX4 | forward: 5’- ACAAGAACGGCTGCGTGGTGAA -3’ |
|  | reverse: 5’- GCCACACACTTGTGGAGCTAGA -3’ |
| LPCAT3 | forward: 5’- CAGGATACCTGGTCTGCTTCCA -3’ |
|  | reverse: 5’- TGAAGAGCCAGTGGATGGTCTG -3’ |
| FTH1 | forward: 5’- TGAAGCTGCAGAACCAACGAGG -3’ |
|  | reverse: 5’- GCACACTCCATTGCATTCAGCC -3’ |
| GSL2 | forward: 5’- TGAGGCACTGTGCTCGGAAGTT -3’ |
|  | reverse: 5’- CCAGTCCAATCAGGCTGGCATT -3’ |
| VDAC3 | forward: 5’- CCATAAACCTTGCTTGGACAGCT -3’ |
|  | reverse: 5’-CATTTCACGCATCTGGCGTTC-3’ |
| LAMP2 | forward: 5’- GGCAATGATACTTGTCTGCTGGC -3’ |
|  | reverse: 5’- GTAGAGCAGTGTGAGAACGGCA -3’ |
| SLC7A11 | forward: 5’- TCCTGCTTTGGCTCCATGAACG -3’ |
|  | reverse: 5’- AGAGGAGTGTGCTTGCGGACAT -3’ |
| ACSL4 | forward: 5’- GCTATCTCCTCAGACACACCGA -3’ |
|  | reverse: 5’- AGGTGCTCCAACTCTGCCAGTA -3’ |
| CARS1 | forward: 5’- CTGGACTACTCCAGCAACACCA -3’ |
|  | reverse: 5’- GACCAGTGATGTCAACAGGAGC -3’ |
| SLC11A2 | forward: 5’-CGCTATTGTCGAGGGAGCTA-3’ |
|  | reverse: 5’-GCTCCACGAAGCTCTTGATG-3’ |
| ACSF2 | forward: 5’-CTCCAGAGTTGATCCGAGCCAT-3’ |
|  | reverse: 5’-CTTCTGCTCCACAGTGTCCTCA-3’ |
| BACH1 | forward: 5’- CACCGAAGGAGACAGTGAATCC -3’ |
|  | reverse: 5’- GCTGTTCTGGAGTAAGCTTGTGC -3’ |
| EPAS1 | forward: 5’- CTGTGTCTGAGAAGAGTAACTTCC -3’ |
|  | reverse: 5’- TTGCCATAGGCTGAGGACTCCT -3’ |
| KEAP1 | forward: 5’- CAACTTCGCTGAGCAGATTGGC -3’ |
|  | reverse: 5’- TGATGAGGGTCACCAGTTGGCA -3’ |
