# Supplementary material for: Phase Separation of NFIB Suppresses SLC3A2‐Mediated Ferroptosis in Castration‐Resistant Prostate Cancer
Source: Adv Sci (Weinh). 2026 Mar 9;13(26):e15340. doi: 10.1002/advs.202515340 (PMC13159144; doi:10.1002/advs.202515340)
Supplement: Supplementary file 3 — Supporting File 3: advs74637‐sup‐0003‐DataFile.zip. [file ADVS-13-e15340-s001.zip › Original images of blots.pdf]

Original Western blots

Order Number	Cell	Protein	Figures
Fig.1H	VCAP, LNCAP, DU145, PC3	SLC3A2	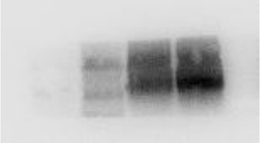
		GAPDH	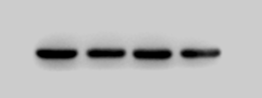
Fig.2A	VCAP, LNCAP, DU145, PC3	NFIB	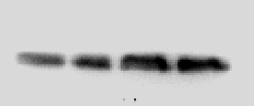
		GAPDH	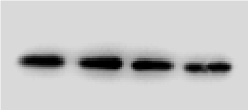
Fig.2D	PC3-sgNFIB	NFIB	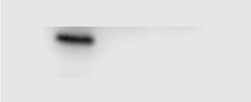
		GAPDH	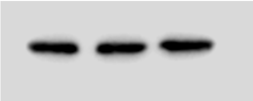
	DU145-sgNFIB	NFIB	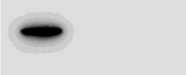
		GAPDH	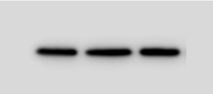
Fig.2J	PC3-OE-NFIB	NFIB	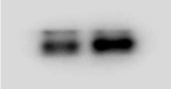
		GAPDH	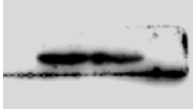
	DU145-OE-NFIB	NFIB	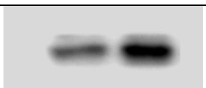
		GAPDH	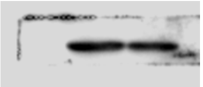
Fig.3D	DU145-sgNFIB	SLC3A2	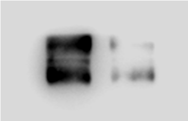
		NFIB	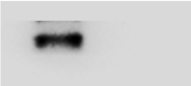

		GAPDH	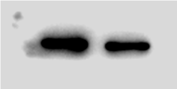
	PC3-sgNFIB	SLC3A2	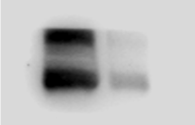
		NFIB	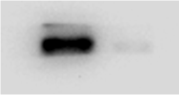
		GAPDH	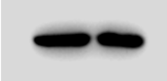
Fig.3M	DU145<br>OE-NFIB<br>+si-SLC3A2	SLC3A2	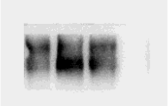
		NFIB	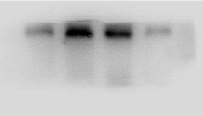
		GAPDH	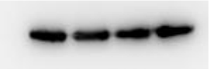
	PC3<br>-OE-NFIB<br>+si-SLC3A2	SLC3A2	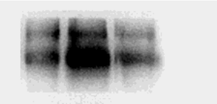
		NFIB	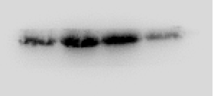
		GAPDH	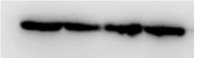
Fig.5E	DU145-sgNFIB<br>(IP)	NFIB	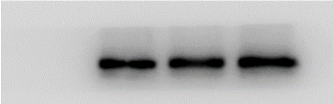
		Ace-lys	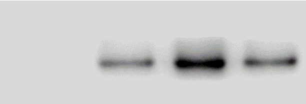
	DU145-sgNFIB<br>(Input)	NFIB	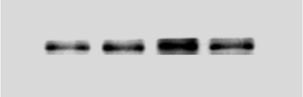
		SLC3A2	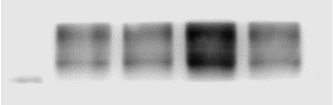
		GAPDH	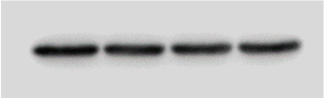

	PC3-sgNFIB (IP)	NFIB	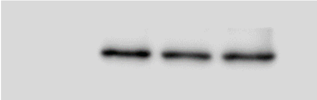
		Ace-lys	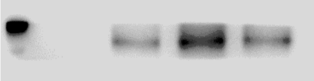
	PC3-sgNFIB (Input)	NFIB	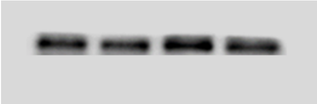
		SLC3A2	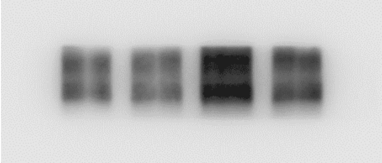
		GAPDH	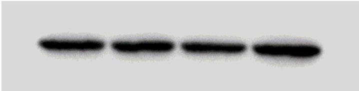
Fig.5F	DU145 (IP)	NFIB	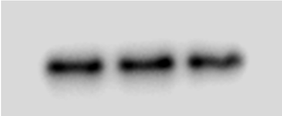
		Ace-lys	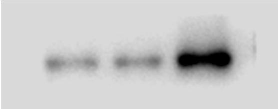
	DU145 (Input)	NFIB	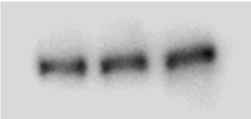
		SLC3A2	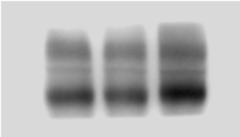
		GAPDH	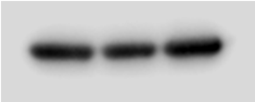
	PC3(IP)	NFIB	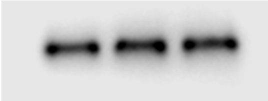
		Ace-lys	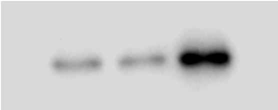
	PC3(Input)	NFIB	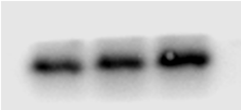
		SLC3A2	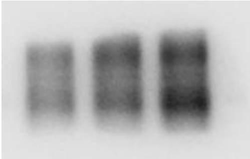

		GAPDH	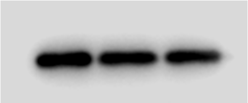
Fig.6A	DU145-sgNFIB	SLC3A2	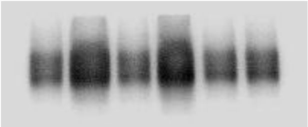
		GFP	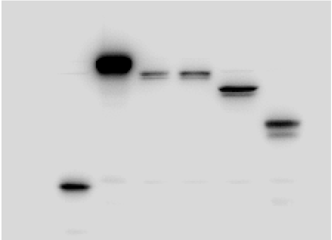
		GAPDH	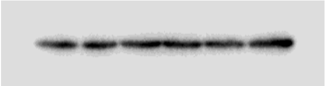
	PC3-sgNFIB	SLC3A2	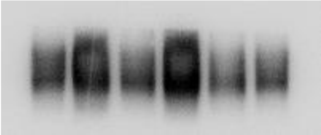
		GFP	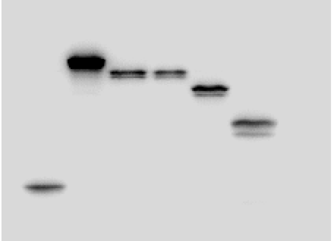
		GAPDH	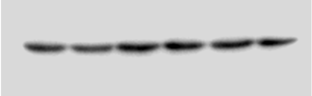
Fig.6E	DU145-sgNFIB (NAM)	NFIB	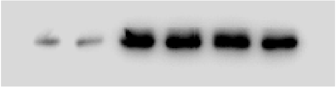
		SLC3A2	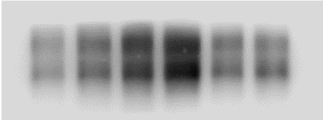
		GAPDH	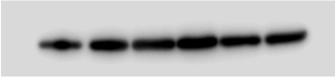
	PC3-sgNFIB (NAM)	NFIB	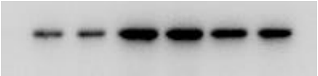
		SLC3A2	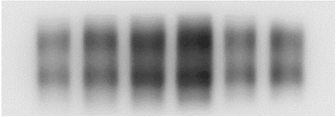
		GAPDH	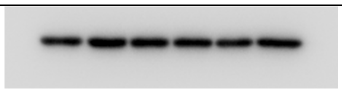

	DU145-sgNFIB (TSA)	NFIB	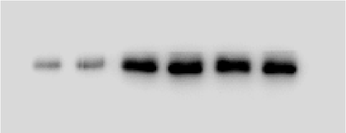
		SLC3A2	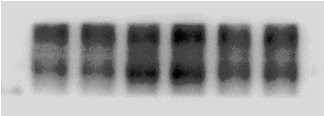
		GAPDH	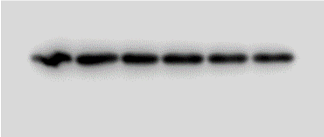
	PC3-sgNFIB (TSA)	NFIB	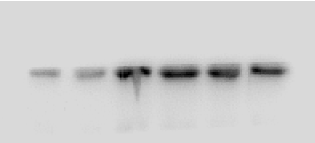
		SLC3A2	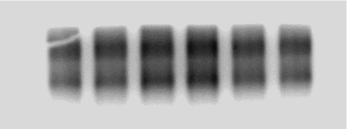
		GAPDH	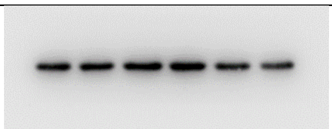
Fig.7A	DU145(IP)	NFIB	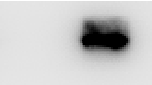
		SIRT1	
		SIRT6	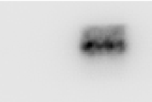
		SIRT7	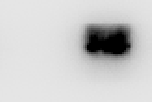
	PC3(IP)	NFIB	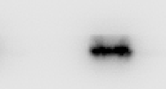
		SIRT1	
		SIRT6	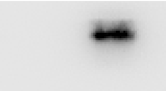

		SIRT7	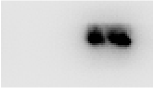
	DU145(Input)	NFIB	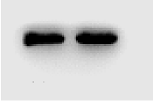
		SIRT1	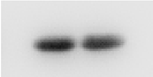
		SIRT6	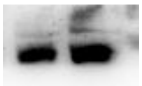
		SIRT7	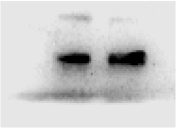
		GAPDH	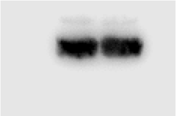
	PC3(Input)	NFIB	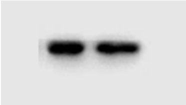
		SIRT1	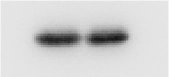
		SIRT6	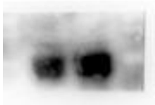
		SIRT7	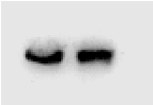
		GAPDH	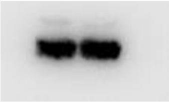
Fig.7B	DU145(IP)	SIRT1	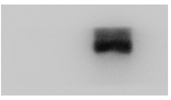
		NFIB	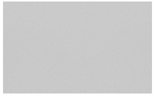
	PC3(IP)	SIRT1	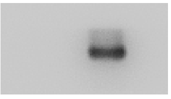
		NFIB	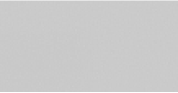

	DU145(Input)	SIRT1	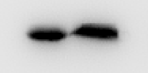
		NFIB	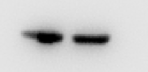
		GAPDH	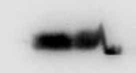
	PC3(Input)	SIRT1	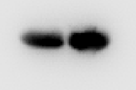
		NFIB	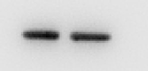
		GAPDH	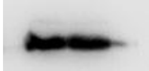
Fig.7C	DU145(IP)	SIRT6	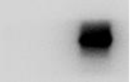
		NFIB	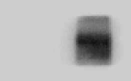
	PC3(IP)	SIRT6	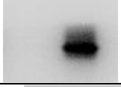
		NFIB	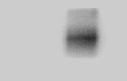
	DU145(Input)	SIRT6	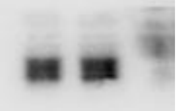
		NFIB	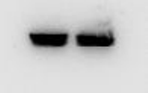
		GAPDH	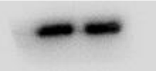
	PC3(Input)	SIRT6	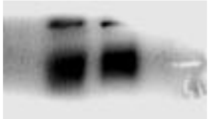
		NFIB	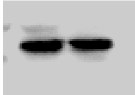
		GAPDH	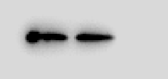
Fig.7D	DU145(IP)	SIRT7	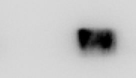
		NFIB	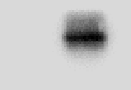
	PC3(IP)	SIRT7	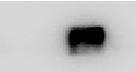

		NFIB	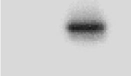
	DU145(Input)	SIRT7	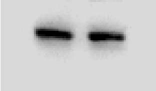
		NFIB	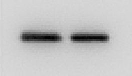
		GAPDH	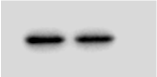
	PC3(Input)	SIRT7	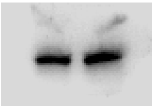
		NFIB	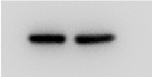
		GAPDH	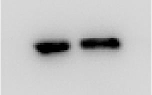
Fig.7E	DU145(IP)	Ace-lys	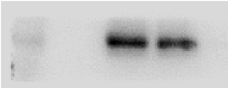
		NFIB	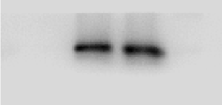
		SIRT6	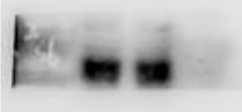
	PC3 (IP)	Ace-lys	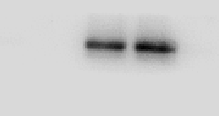
		NFIB	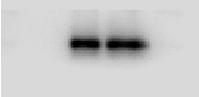
		SIRT6	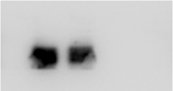
	DU145 (Input)	NFIB	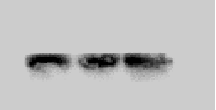
		SLC3A2	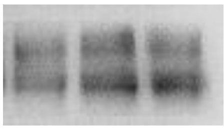
		GAPDH	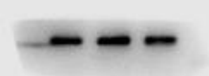

	PC3(Input)	NFIB	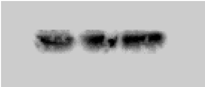
		SLC3A2	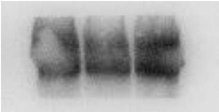
		GAPDH	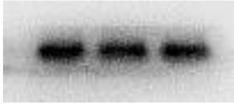
Fig.7F	DU145(IP)	Ace-lys	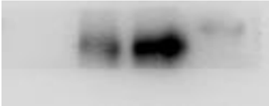
		NFIB	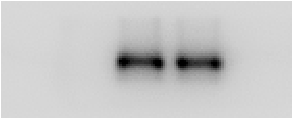
		SIRT7	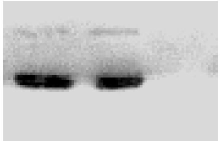
	PC3 (IP)	Ace-lys	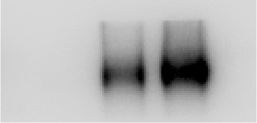
		NFIB	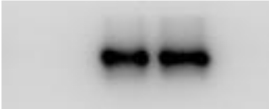
		SIRT7	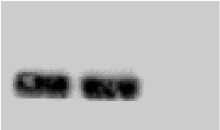
	DU145 (Input)	NFIB	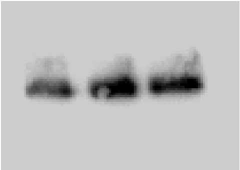
		SLC3A2	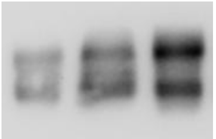
		GAPDH	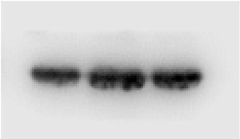

	PC3(Input)	NFIB	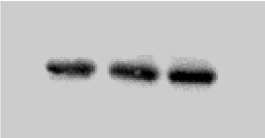
		SLC3A2	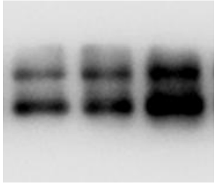
		GAPDH	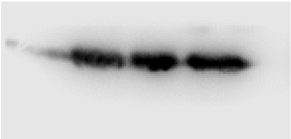
Fig.S3B	DU145-sgNFIB (LLPS)	PARP	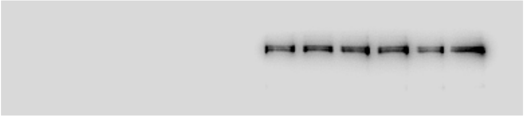
		GAPDH	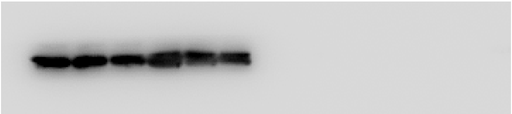
		GFP	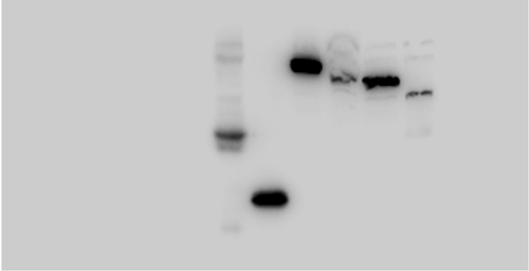
	PC3-sgNFIB (LLPS)	PARP	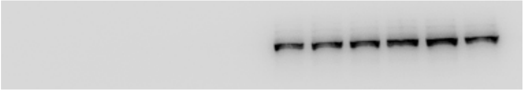
		GAPDH	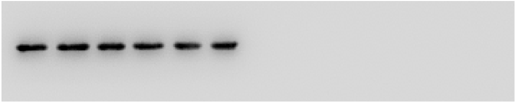
		GFP	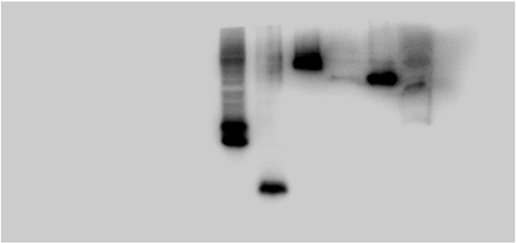
Fig.S3D	DU145 (IP)	NFIB	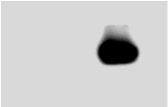

		Ace-lys	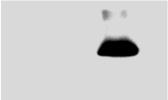
	DU145 (Input)	NFIB	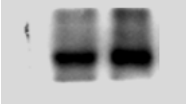
		GAPDH	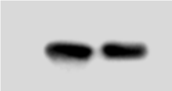
	PC3(IP)	NFIB	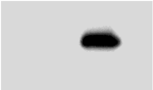
		Ace-lys	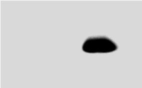
	PC3(Input)	NFIB	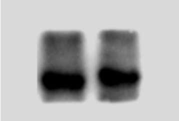
		GAPDH	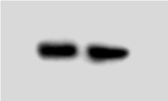
