# Supplementary material for: Phase Separation of NFIB Suppresses SLC3A2‐Mediated Ferroptosis in Castration‐Resistant Prostate Cancer
Source: Adv Sci (Weinh). 2026 Mar 9;13(26):e15340. doi: 10.1002/advs.202515340 (PMC13159144; doi:10.1002/advs.202515340)
Supplement: Supplementary file 3 — Supporting File 3: advs74637‐sup‐0003‐DataFile.zip. [file ADVS-13-e15340-s001.zip › Supplemental information 1.pdf]

Supplemental Information 1

A

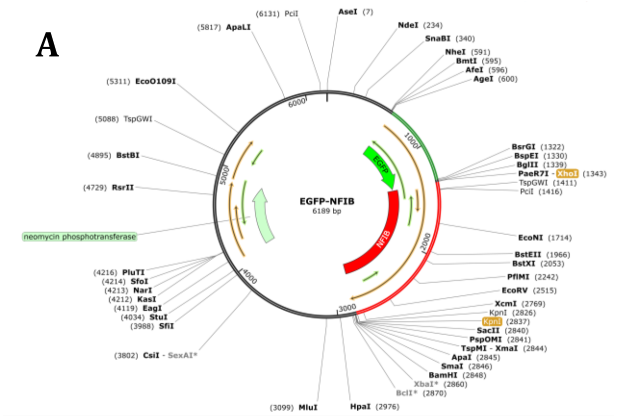

B

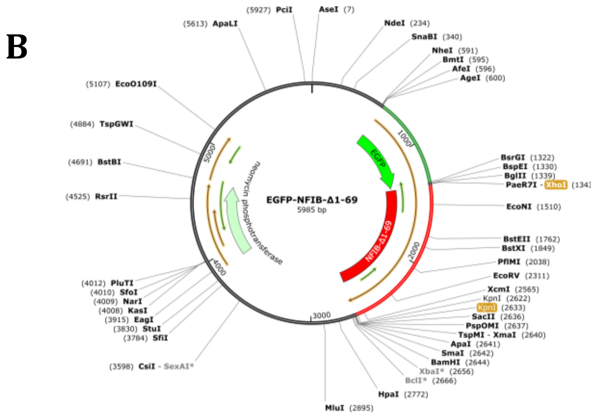

C

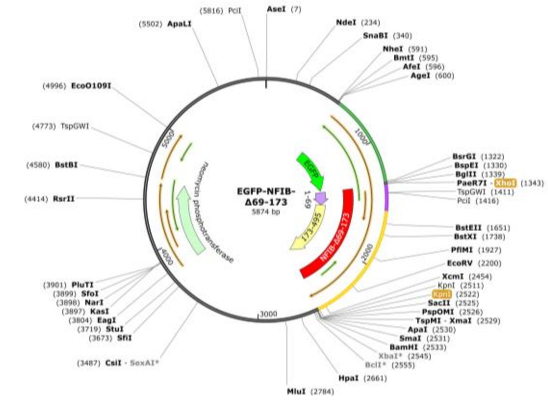

D

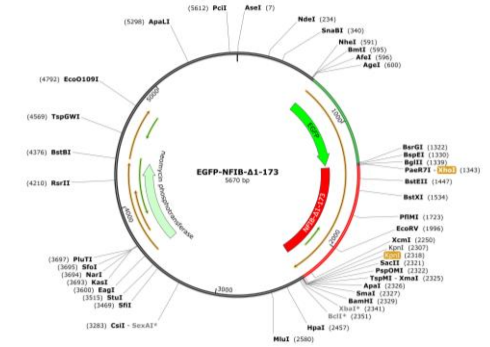

E

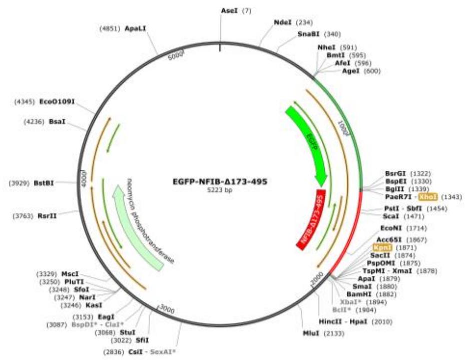

F

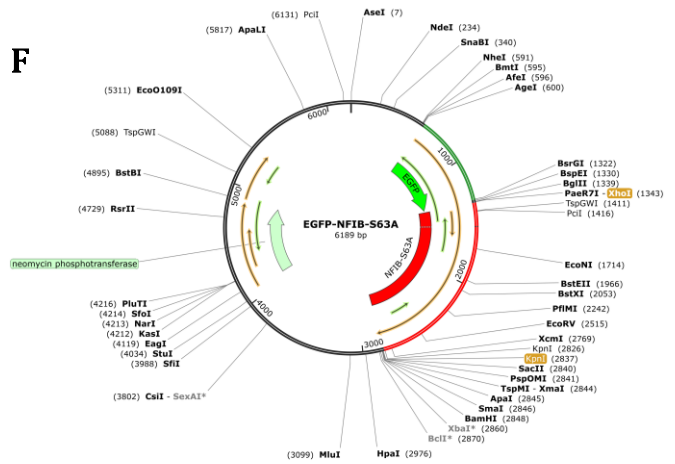

G

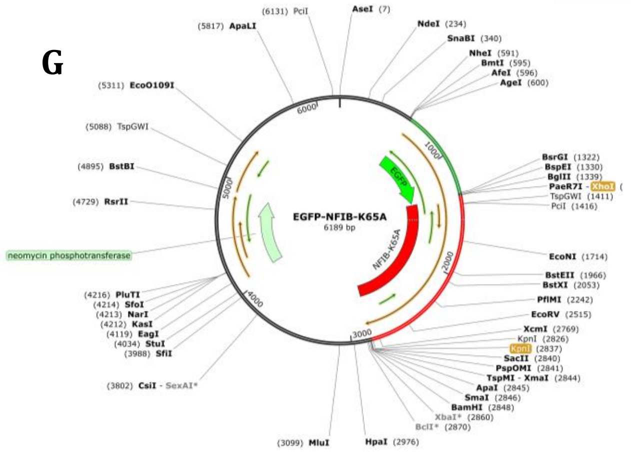

H

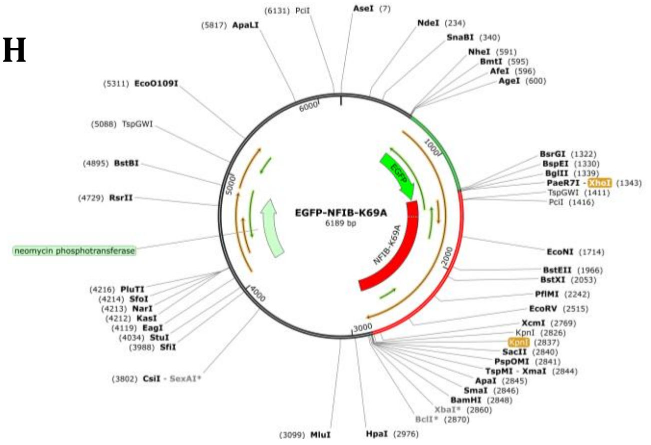

Schematic representation of the sequences of NFIB and its mutant plasmids.

(A) Schematic representation of the EGFP-NFIB plasmid and its restriction enzyme map.

(B-E) Plasmid map of deletion mutations in phase separated segment of NFIB.

(F-H) Plasmid map of NFIB point mutations.
