# Supplementary material for: Phase Separation of NFIB Suppresses SLC3A2‐Mediated Ferroptosis in Castration‐Resistant Prostate Cancer
Source: Adv Sci (Weinh). 2026 Mar 9;13(26):e15340. doi: 10.1002/advs.202515340 (PMC13159144; doi:10.1002/advs.202515340)
Supplement: Supplementary file 3 — Supporting File 3: advs74637‐sup‐0003‐DataFile.zip. [file ADVS-13-e15340-s001.zip › Supplemental information 2.pdf]

Supplemental Information 2

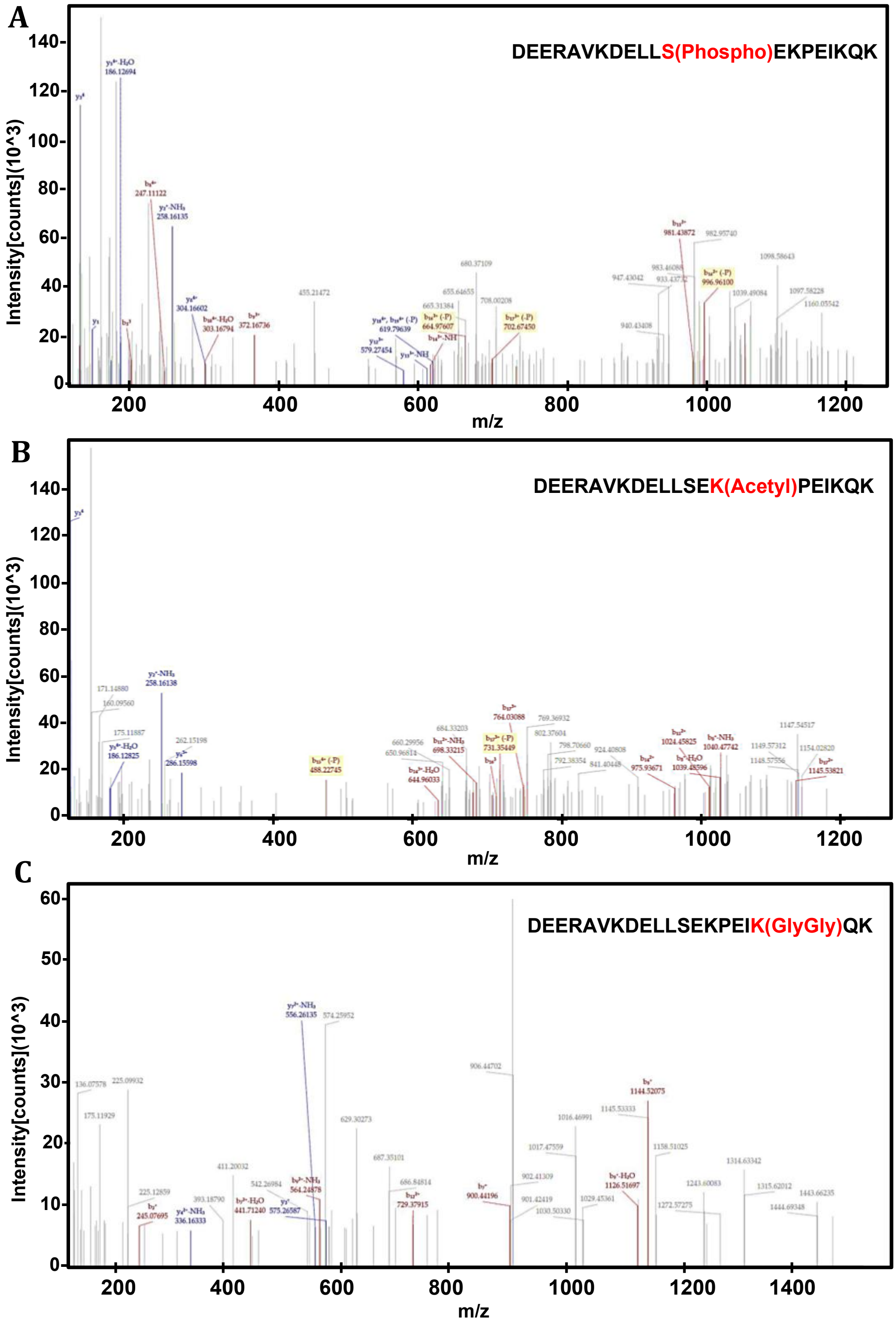

Mass spectrometry analysis of NFIB identified residues Ser63, Lys65, and Lys69 as the NFIB sites for phosphorylation, acetylation and ubiquitination, respectively.
